# How many species and under what names? Using DNA barcoding and GenBank data for west Central African amphibian conservation

**DOI:** 10.1371/journal.pone.0187283

**Published:** 2017-11-13

**Authors:** Jessica L. Deichmann, Daniel G. Mulcahy, Hadrien Vanthomme, Elie Tobi, Addison H. Wynn, Breda M. Zimkus, Roy W. McDiarmid

**Affiliations:** 1 Center for Conservation and Sustainability, Smithsonian Conservation Biology Institute, National Zoological Park, Washington, DC, United States of America; 2 Global Genome Initiative, National Museum of Natural History, Smithsonian Institution, Washington, DC, United States of America; 3 Department of Vertebrate Zoology, Division of Amphibians and Reptiles, National Museum of Natural History, Smithsonian Institution, Washington, DC, United States of America; 4 Museum of Comparative Zoology, Harvard University, Cambridge, MA, United States of America; 5 USGS, Patuxent Wildlife Research Center, National Museum of Natural History, Washington DC, United States of America; Universitat Trier, GERMANY

## Abstract

Development projects in west Central Africa are proceeding at an unprecedented rate, often with little concern for their effects on biodiversity. In an attempt to better understand potential impacts of a road development project on the anuran amphibian community, we conducted a biodiversity assessment employing multiple methodologies (visual encounter transects, auditory surveys, leaf litter plots and pitfall traps) to inventory species prior to construction of a new road within the buffer zone of Moukalaba-Doudou National Park, Gabon. Because of difficulties in morphological identification and taxonomic uncertainty of amphibian species observed in the area, we integrated a DNA barcoding analysis into the project to improve the overall quality and accuracy of the species inventory. Based on morphology alone, 48 species were recognized in the field and voucher specimens of each were collected. We used tissue samples from specimens collected at our field site, material available from amphibians collected in other parts of Gabon and the Republic of Congo to initiate a DNA barcode library for west Central African amphibians. We then compared our sequences with material in GenBank for the genera recorded at the study site to assist in identifications. The resulting COI and 16S barcode library allowed us to update the number of species documented at the study site to 28, thereby providing a more accurate assessment of diversity and distributions. We caution that because sequence data maintained in GenBank are often poorly curated by the original submitters and cannot be amended by third-parties, these data have limited utility for identification purposes. Nevertheless, the use of DNA barcoding is likely to benefit biodiversity inventories and long-term monitoring, particularly for taxa that can be difficult to identify based on morphology alone; likewise, inventory and monitoring programs can contribute invaluable data to the DNA barcode library and the taxonomy of complex groups. Our methods provide an example of how non-taxonomists and parataxonomists working in understudied parts of the world with limited geographic sampling and comparative morphological material can use DNA barcoding and publicly available sequence data (GenBank) to rapidly identify the number of species and assign tentative names to aid in urgent conservation management actions and contribute to taxonomic resolution.

## Introduction

Species lists are necessary for conservation planning and management [1]. As such, conservation biologists and ecologists must provide taxonomic information to decision-makers for conservation efforts to move forward. Unfortunately, taxonomy has not kept pace with habitat loss in high biodiversity areas [2], and a common criticism of studies in conservation biology and ecology are that they lack taxonomic rigor [2, 3]. Put simply, there is an urgent need for taxonomic names and counts of species, but assigning definitive names and distinguishing closely related species is problematic, more so for some groups than others.

In Central Africa, as in many other parts of the world, economic development is putting increased pressure on biodiversity [4]. Expansion of logging [5], road development [6], agriculture [7], oil and gas development [8], and other forms of energy development are causing changes in land use patterns and consequently affecting biotic communities. Spending on infrastructure in Africa between 1998 and 2007 increased annually by 17 percent and that growth is expected to accelerate [9]. It is estimated that up to $450 billion will be invested in energy infrastructure development and production in sub-Saharan Africa over the next few decades [10]. Consequently, Environmental Impact Assessments (EIAs) are routinely being carried out in many African nations to evaluate potential effects of various proposed development projects on the environment.

Expected changes in land use combined with projected climate change are likely to have particularly negative consequences for tropical African amphibian biodiversity [11]. Amphibians play a crucial role in properly functioning ecosystems, contributing to nutrient cycling, bioturbation, energy flow, food webs, and other ecosystem dynamics [12, 13]. These animals provide additional ecosystem services valuable to humans, such as regulating pests, acting as a food source, functioning as models for medical research and serving as subjects of recreational and spiritual importance that vary across cultures [13, 14]. Given their critical importance in functional ecosystems, maintenance of amphibian diversity in the face of anthropogenic change is essential. However, in order to be able to maintain amphibian diversity, we must first accurately measure it so that changes can be documented and restoration measures implemented in disturbed systems.

Unfortunately, the issue of determining the total number of species of amphibians in a given area is not a simple task. In spite of the fact that amphibians are the most threatened group of vertebrates to be assessed to date [15, 16], species of this class are also among the most poorly known vertebrate groups in many geographic areas (e.g. [17]). In the Afrotropics in particular, the lack of basic knowledge regarding amphibian species taxonomy and richness and high numbers of undescribed species make the task of surveying and identifying amphibians more difficult [18]. Although recent taxonomic field guides have improved (e.g. [19]), anurans include groups rife with cryptic species [20], and others with high levels of intraspecific phenotypic polymorphism exist [21, 22]. These factors complicate not only specimen identification, but also simple documentation of the number of species at a given site. Confounding matters even further, expertise in amphibian taxonomy is limited globally and tends to be restricted to countries with high economic income [23]. In many cases, basic biodiversity assessments in developing countries are carried out by parataxonomists, invaluable field personnel charged with sorting specimens into taxonomic units, who vary in training and ability to evaluate diagnostic morphological traits [24–27]. Making matters worse, published keys meant to aide in identification can be unclear, confusing, or in need of improvement because many problematic groups have not been worked out taxonomically.

Integrating additional tools into traditional morphological species inventories can contribute a great deal to the accuracy and precision of species richness estimates in an area [28–30]. DNA barcoding is one such tool that can be added at relatively limited costs and presents non-taxonomists with a way to verify and improve identifications and species diversity estimates [31–33]. DNA barcoding is likely to be a very useful tool in species monitoring, particularly for taxa that can be difficult to distinguish based on morphology alone (i.e. cryptic species). In addition, DNA barcoding can also reduce errors in identifying species with high phenotypic variability and polymorphisms, which is less frequently discussed in the literature when compared to its potential to aid in cryptic species identification.

The advent of DNA barcoding has opened up new potential solutions to the issue of characterizing diversity; however, this method requires careful interpretation when used to assign names to groups of similar specimens (i.e. clusters or operational taxonomic units [OTU]) [34], particularly with amphibians [35]. When a DNA barcode library exists for comparisons of specimens from the field, it can be used to identify specimens to a species [36]. In fact, the existence of DNA barcode libraries has proven useful for a variety of biodiversity assessments (e.g. [32, 33, 37, 38]) and ecological studies [39, 40] representing different taxonomic groups. However, given that the intent of DNA barcoding is not to reconstruct the evolutionary history of a lineage [36], the usefulness of a DNA barcode library for assigning names to OTUs is heavily dependent on the accuracy of reference sequences. Misidentified and consequently mislabeled specimens and their associated sequence data can hinder the ability to identify specimens accurately.

Here we report the results of an integrated amphibian survey in an area in southwestern Gabon prior to the construction of a road. Our primary goal was to document amphibian species richness within the study area so that appropriate conservation management measures could be taken during and after development. We use the results of our DNA barcoding analyses to identify specimens, thereby refining our initial field identifications based on morphology, followed by systematic comparisons of our genetic material to sequences in GenBank, morphological comparisons to additional materials available in the National Museum of Natural History (NMNH), and the primary literature, including recent work and original species descriptions. By doing so, we contribute more widely to a DNA barcode library for amphibians of this region and summarize the current knowledge of west Central African anuran taxonomy by including genetic material of specimens available to us at the NMNH.

We present this as a model study for use by parataxonomists, ecologists and conservation biologists needing to rapidly define taxonomy of problematic groups using DNA barcoding and limited comparative material. Our study demonstrates how additional taxonomic expertise and comparative material can be used for problematic groups. In parallel, we offer advice to taxonomists who have more comparative material and additional resources available to undertake future comprehensive taxonomic treatments of these groups. Through this west Central African case study, we provide methodological insights that, if implemented, will better integrate biodiversity inventories and taxonomy for the benefit of biodiversity conservation.

## Materials and methods

### Study area

**Fig 1:**
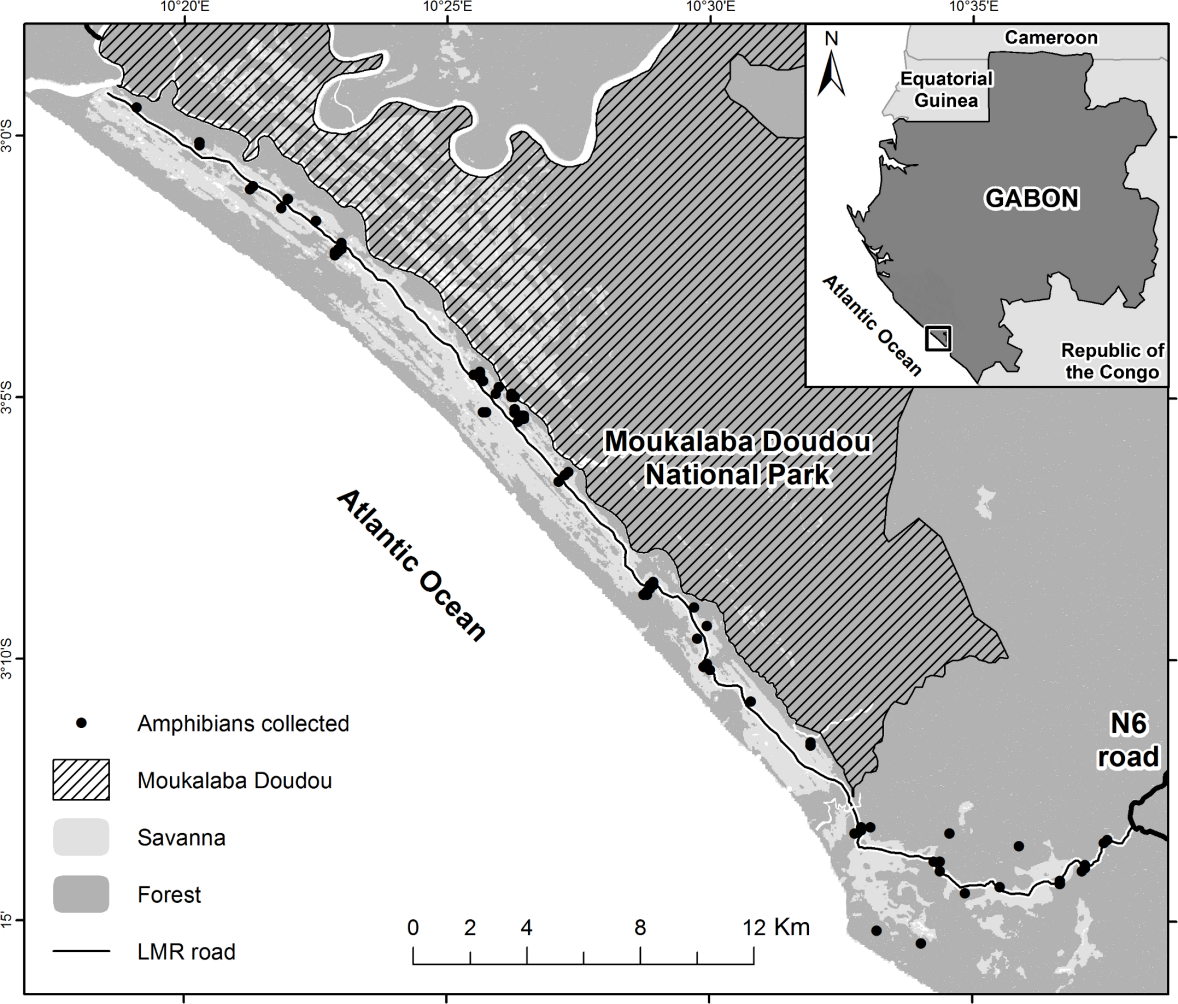
Loubomo-Mougagara Road (LMR) study area for amphibian biodiversity inventory in Nyanga province, Gabon.

The study site lies along the Atlantic coast of Gabon in the Nyanga Province and includes a mosaic of grassland, secondary forests, and wetlands covering approximately 175 km^2^ (Fig 1). Annual rainfall averages roughly 2,000 mm. A short dry season occurs in January with a longer dry season from late May to September. The research area is a 52 km-long band centered on the projected path of the Loubomo-Mougagara Road (LMR) and extending 2–3 km on either side. Moukalaba-Doudou National Park borders most of the research area to the northeast, and the eastern end of the LMR path terminates at the N6 road. The area is bordered to the south and west by the Atlantic Ocean. In 2014 (after biodiversity surveys), construction of the two-lane LMR began and the laterite phase of the road was completed in 2016.

### Field sampling

We sampled amphibians between May 20–June 19, 2012 (early dry season) and March 13–April 23, 2013 (short wet season 2013). We used a variety of sampling methods, including nocturnal and diurnal visual and acoustic surveys along 22 pre-determined and randomly selected 500 m transects in all habitat types (forests, savannas, swamp margins), pitfall traps near habitat transition zones, and opportunistic visual encounters.

In the dry season, the research team consisted of a parataxonomist (ET) previously trained in amphibian taxonomy, a student, and a field assistant. In the wet season, the team was joined by a lab assistant and a more experienced amphibian biologist (JLD), albeit a novice in African amphibian taxonomy. We used published assessments and species lists of amphibians of Gabon [41–43] and a field guide [19] to identify specimens in the field. Vouchers were collected for all morphologically recognized species (morphospecies), and some were also photographed. Specimens were euthanized in the field using 20% benzocaine gel (Oragel) applied to the ventral surface of the body [44]. After euthanasia, a sample of thigh muscle was taken and the tissue stored in 95% ethanol. Voucher specimens were subsequently preserved in 10% formalin and stored in 70% ethanol. Specimens were deposited in the collection of the United States National Museum (USNM) housed at the NMNH, Smithsonian Institution, Washington, DC. In order to confirm species identifications made in the field, we barcoded 80 specimens from the first (dry) season and 97 specimens from the second (wet) sampling season for a total of 177 specimens from the LMR study area.

### Ethics statement

LMR specimens were collected under research permits AR0016/12, AR0004/13 and 00170MEF from the Centre National de la Recherche Scientifique et Technologique, the Agence Nationale des Parcs Nationaux, and the Direction de l’Aménagement des Aires Protégées of Gabon. The LMR field study was carried out in strict accordance with the guidelines set forth by the American Society of Ichthyologists and Herpetologists (ASIH), the Herpetologists' League (HL), and Society for the Study of Amphibians and Reptiles (SSAR). The LMR specimen euthanasia method has been approved by the above institutions as well as the American Veterinary Medical Association [44], and all efforts were made to minimize suffering. The protocol was approved by the Smithsonian National Zoological Park’s Institutional Animal Care and Use Committee (NZP-IACUC permit #12–13).

### DNA barcode protocol

Extractions of genomic DNA were performed on an AutoGenprep 965 (2011 AutoGen, Inc.), using the manufacturer's standard phenol protocol. Genomic DNA was eluted in 100μl of AutoGen R9 re–suspension buffer. Polymerase chain reactions (PCR) were conducted for the mtDNA large ribosomal subunit (rrnL: 16S) and cytochrome oxidase subunit I (COI) using the primers: 16Sar 5' CGCCTGTTTATCAAAAACAT 3' and 16Sbr 5' CCGGTCTGAACTCAGATCACGT 3' [45] and COI–fishCO1F 5' TCAACYAATCAYAAAGATATYGGCAC 3' and fishCO1R 5' ACTTCYGGGTGRCCRAARAATCA 3' [46] or Chmf4 5' TYTCWACWAAYCAYAAAGAYATCGG 3' and Chmr4 5' ACYTCRGGRTGRCCRAARAATCA 3' [47]. PCR conditions were performed in 10μl reactions following the *3*.*6 PCR Methods*: *Amplification* protocol of Weigt et al. [48] with annealing temperatures of 54°C for 16S and 48°C for COI. Sequence reactions were performed in both directions with the PCR primers using BigDye^®^ Terminator v3.1 Cycle Sequencing Kit’s in 0.25x 10μl reactions and run on an Automated ABI 3730 Sequencer (2011 Life Technologies). Raw chromatograms were edited in Sequencher^®^ v5.1 (2012 Gene Codes Corp.), complementary strands were aligned, and COI was inspected for proper translation, using the DNA barcode trim criteria described in Weigt et al. [48]. Alignments for both genes were conducted using the MAFFT plug-in in Geneious v8.1.7 with default settings. We chose to characterize a portion of the 16S gene in addition to producing COI barcodes for our specimens because we anticipated needing to compare our data to the large amount of 16S sequence data available in GenBank for west Central African anurans.

### Additional reference material

**Fig 2:**
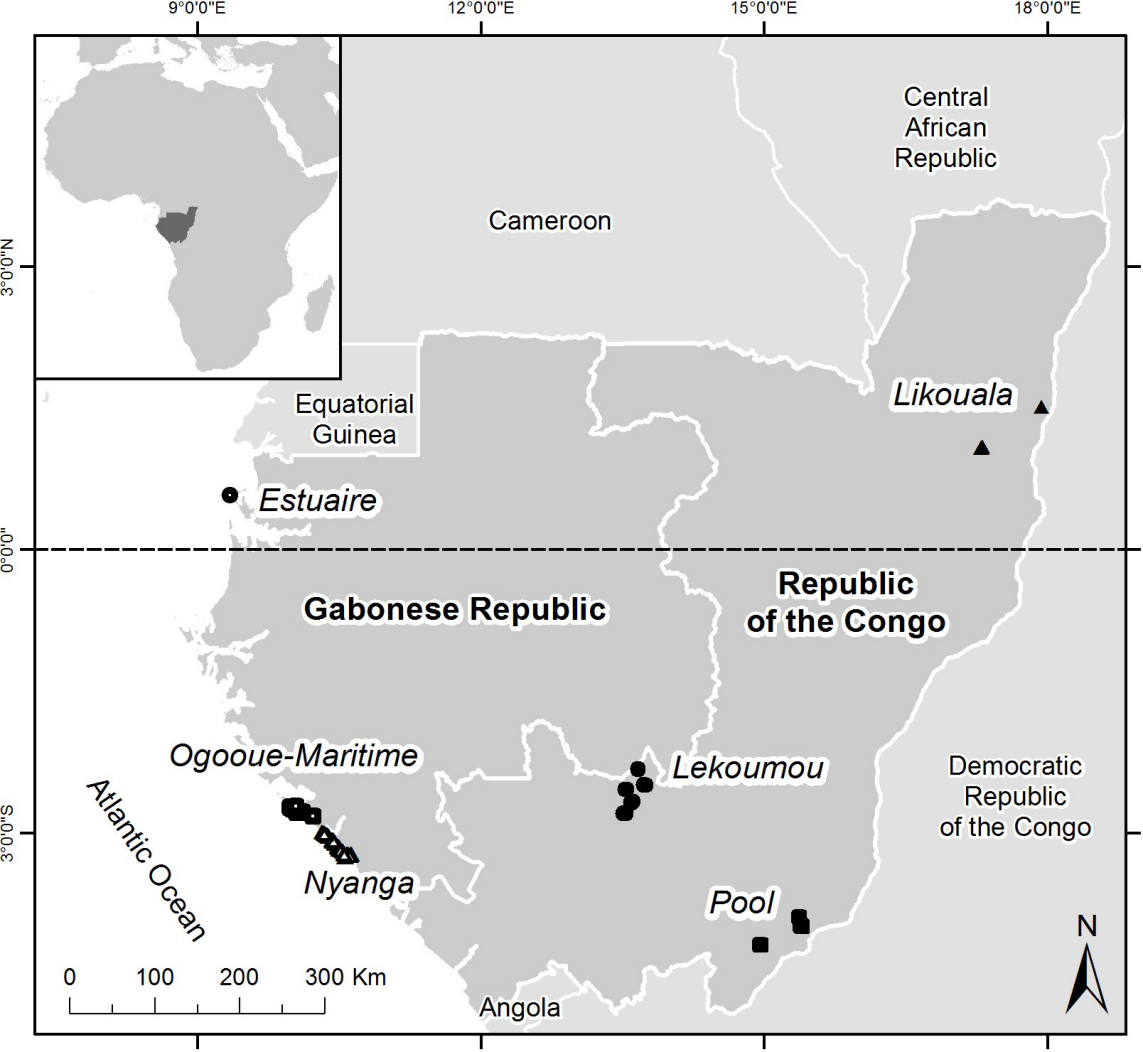
Reference map showing localities of all barcoded material. White circle: Estuaire province (Gabon), white squares: Ogooue-Maritime province (Gabon), white triangles: Nyanga province (Gabon; LMR study site), black circles: Lekoumou department (Republic of Congo, RC), black squares: Likouala department (RC), and black triangles: Pool department (RC).

In order to aid in identification of species from the LMR study site, we barcoded additional reference specimens from the USNM collection. We attempted to sample all species known to occur in Gabon, as well as some museum specimens collected in neighboring Republic of Congo (RC), for barcode analyses (Fig 2). In addition to the material collected from the LMR study site, we barcoded 90 specimens collected previously from sites in Estuaire and Ogooue-Maritime provinces in Gabon (approximately 400 and 25 km from the LMR site, respectively), and 275 amphibian specimens collected from four sites in the RC (Fig 2). All material is cataloged and housed in the USNM collection. Tissues from these specimens were initially placed in 95% ethanol and subsequently stored at -80°C after deposition in the USNM collection. Some voucher specimens, including those from the LMR site (N = 2), other Gabonese sites (N = 3), and Congolese sites (N = 7), have been lost or destroyed. For some of those specimens, photographs serve as the voucher and are referred to as USNM Herp Image Number (N = 3); the other tissue samples without photographic vouchers are catalogued as USNM Herp Tissue Number (N = 9).

In addition to using available museum specimens, we also queried the Barcode of Life Database (BOLD) and GenBank for sequences from all amphibian species known to occur in region (as of 11 November 2016) and for all genera for which we generated sequence data for comparative purposes.

### Data analysis

Researchers use a variety of methods for analyzing DNA barcode data, including percent sequence divergence [49], neighbor-joining (NJ) trees [36], the Barcode Index Number (BIN) System [50], and haplotype networks [51] among other methods. Given our level of taxonomic and geographic sampling, we chose a variety of methods to analyze our data including percent sequence divergence, neighbor-joining trees, BINs, and the Automatic Barcode Gap Discovery (ABGD) method [52].

For COI data, we first used the Barcode Index Number (BIN) System [50] generated in BOLD for specimen identification. However, because the available DNA barcode library for anurans is limited, we could not rely solely on this method. For example, a search in GenBank (Dec. 2015) for ‘Anura’ and ‘Africa’ reveals 170 COI records (120 of which were from frogs of the genus *Xenopus*). In addition to the BINs method, we incorporated an alternative assessment of specimen identification using the Automatic Barcode Gap Discovery (ABGD) method [52] with our COI data. The ABGD method uses adjustable parameters for group sequences (we used Pmin-0.001, Pmax = 0.1, 10 steps, X = 1.5, and Nb = 20), rather than a fixed difference (e.g. >2.2%; BIN). We directly compared the results of these two methods, whereas others discuss their differences in more detail [52]. We also generated Neighbor–joining (NJ) trees in PAUP* v4.0b10 [53] for the 16S and COI data separately, and the two loci were concatenated for all the sequences we generated in a combined Maximum Likelihood analysis using RAxML [54] and the GTRGAMMA model (partitioned by gene) with simultaneous fast bootstrap method (100 replicates) and best ML tree search. We then conducted neighbor-joining tree searches in PAUP* with the GenBank 16S material to assess sequence similarity (see below). We acknowledge that NJ trees are not ideal for reconstructing evolutionary history, particularly with the small sized DNA sequences of COI or 16S. However, we use them as a useful tool for clustering similar sequences of putative members of the same species, not for estimating evolutionary relationships. We did not use haplotype networks because they work best when taxonomic and geographic sampling are most complete, which is not the case in our study. Most of our specimens per species are from effectively only one or two localities whereas the species likely occur all throughout the region.

### Taxonomic assignments

We attempted to assign each specimen to a putative species given the results of various lines of evidence (see below). If species assignment was not possible, we assigned a temporary species name (e.g. sp. A) to a given specimen or groups of specimens that we think represent the same undescribed species, i.e. “known unknowns” [36]. We consider this a process of “species discovery”[36], were conservative in our assignments of specimens to these categories, and we followed the general lineage concept of species [55] using the criteria defined below.

As a result of a paucity of existing records of anuran COI barcodes in BOLD, we were unable to rely solely on the BINs method to verify identifications of our specimens; therefore, we determined the number of species represented by our specimens and their taxonomy based on multiple lines of evidence: 1) COI BINs calculated in BOLD and the ABGD method; 2) 16S sequences compared with GenBank material; 3) traditional morphological species descriptions, including comparisons to type descriptions, assessments of proximity to type localities, and known geographic distributions of the putative species in question (for further information, see below).

#### COI BINs

If specimens were placed in the same COI BIN in BOLD and in a single ABGD group, they were considered the same species. If specimens initially identified as the same species were placed in separate BINs or ABGD groups, they were further evaluated. We directly compared the results of the ABGD method with the BINs. If the separate BINs or ABGD groups included specimens from different geographic locations, and the 16S data placed them in the same clade, they were treated as the same species with genetic variation assumed to be based on geography. If specimens from the same locality were placed in separate BINs or ABGD groups and the 16S data placed them in the same clade, they were treated as the same species with population-level genetic variation. If specimens from the same locality were placed in separate BINs or ABGD groups and the 16S data placed them in different clades, they were treated as separate species.

#### 16S sequences

For these samples we used criteria similar to our COI BINs and ABGD groups method described above, and treated 16S clades similar to COI BINs and ABGD groups. We aligned all of the 16S sequences from GenBank with our sequences using MAFFT in Geneious (default settings), and generated NJ trees in PAUP*. Specimens identified as the same species and in the same clade, including our sequences and those from GenBank, were treated as the same species. Specimens identified as the same species but in different clades and different localities but monophyletic (i.e. sister to each other) were treated as geographic variation of the same species if they fit the morphological description of that species (see below). If they did not fit the morphological description of that species or showed substantial genetic variation, they were treated as different species. Specimens morphologically identified as the same species but in different clades, sister to other species were treated as different species.

#### Morphological species descriptions

Given the taxonomic scope of our project, the timeliness of the project in conjunction with specimens currently on loan to other researchers, and the fact that many GenBank voucher specimens are difficult to track down, were unable to examine the morphology of all GenBank vouchers. It is likely that parataxonomists working in developing countries would also have difficulty in such an undertaking. Therefore, we offer this more rapid cursury method as an intermittent solution to the task of proper amphibian taxonomy. Additionally, we could not rely entirely on species identifications in GenBank, we realized inconsistent and inaccurate identifications could affect all specimens (from the LMR study site, reference material, and other GenBank sequences). Accordingly, we attempted to verify the identity of the LMR site specimens by comparing them to morphological descriptions of species, including original descriptions and subsequent works, and taking into account the geographic distance between our specimens and the GenBank records, as well as the genetic distances between sequences. Unfortunately, no method or algorithm takes all of these factors into account to determine species designations automatically. Therefore, we relied on our taxonomic expertise regarding anuran diversity in this region. In so doing, we attempted to be conservative, recognizing widespread, genetically variable species (rather than identifying each geographic clade as a distinct species), and point out cases where additional sampling, data collection, and analyses are needed to investigate further what may represent greater diversity than previously recognized in such groups.

We acknowledge that this method is overly simplistic, and true species boundaries may be masked by factors such as hybridization, incomplete lineage sorting, and geographic structure inconsistent with taxonomy. However, we propose this as a method for parataxonomists working in developing countries to obtain accurate species inventories, which may also bridge conservation ecologists and taxonomists in further sorting out west Central African amphibian taxonomy through supplemented geographic and taxonomic coverage (demonstrated on amphibians here, but may also be applied to other taxonomic groups).

### Community species richness analysis

Using the results of field (in-situ and morphologically-based) identifications and of DNA barcode identifications of amphibian specimens collected in the LMR study area during each sampling period (dry and wet seasons) and overall, we employed EstimateS [Version 9.1, 56] to calculate sample-based rarefaction curves for species richness and to calculate the non-parametric incidence-based species richness estimator, Chao2 [57] for each identification method and in each season. We used sample-based rather than individual-based rarefaction because reference material was not collected for all individuals encountered in the field; therefore, any abundance- or individual-based richness estimates would be inaccurate. Richness estimates for the LMR study site presented here are based solely on data from collected material. To determine statistical significance of rarefied species richness, we compared overlap of 84% (P = 0.05) confidence intervals of the accumulation curves for each season [58, 59].

## Results

### Molecular data

**Fig 3:**
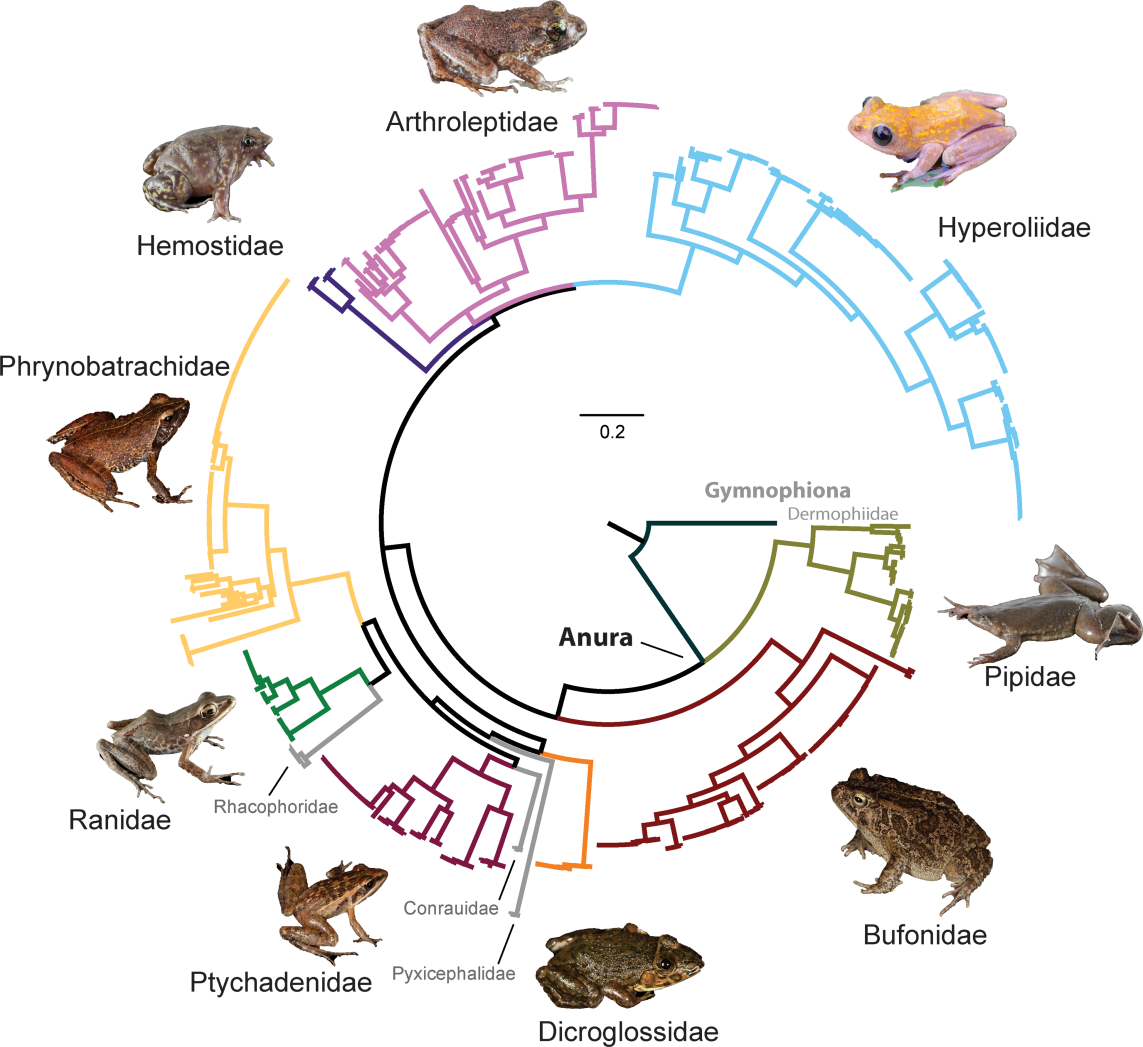
Maximum-likelihood phylogeny of the combined 16S and COI DNA barcode data for all specimens sequenced for this study. Family clades observed at our study site are color-coded and show a representative image. The four families shown in grey (including the caecilian) were not observed at the Loubomo-Mougagara Road study site but were included as reference material from other sites in Gabon or the Republic of Congo.

We characterized DNA sequence data from 540 specimens, including 524 COI DNA barcode sequences ranging from 498–654 base-pairs (bp) obtained and placed in 94 BINs in BOLD and 85 ABGD groups, and 528 16S sequences ranging from 493–579 bp (Table 1). Our material included one caecilian specimen (Gymnophiona) and 539 specimens from 12 families, 21 genera, and 72 species of anurans. Our concatenated alignment of COI sequences was 654 bp, and the 16S alignment was 655 bp. Our 16S and COI sequences have been deposited in GenBank under the accession numbers KY079387–KY080438, and we included trace files to obtain the Keyword "Barcode" in GenBank for all COI sequences. All COI and 16S records have also been deposited in BOLD (Process IDs: WAFRA001-14 to WAFRA542-14; West Central African amphibian barcode library for biodiversity). Our concatenated (COI and 16S) maximum-likelihood phylogeny of the DNA barcoded specimens shows families observed at our study site and additional families sampled in our reference material from elsewhere in Gabon and the RC (Fig 3). A list of species identified at the LMR study site, elsewhere in Gabon, and from the RC is shown in Table 2 along with a direct comparison of BINs and ABGD groups. All discrepancies between the BINs and ABGD groups are restricted to instances where a single species consisted of multiple BINs. Species were placed into 2–3 BINs, but only in one ABGD group in seven cases; two of these occurred where there were 3 BINs per species (*Hyperolius olivaceus* and *H*. *adspersus*). In one case, *Phyrnobatrachus auritus* was placed in three BINs and two ABGD groups. These cases resolve the difference between the number of BINs and ABGD groups. Interestingly, eight cases occurred where species put into multiple BINs were equally divided among ABGD groups (Table 2). In all other cases, the two methods agreed. Below we present species accounts for specimens identified in our study, organized taxonomically by order and family. The associated NJ trees can be found in Supporting Information (S1–S9 Figs). We discuss relevant GenBank sequences that are potentially misidentified.

**Table 1:** Number of sampled specimens and COI and 16S sequences successfully amplified from specimens at each collection site.

Collection Locality	Total Number of Specimens	Number of COI Sequences	Number of 16S Sequences
LMR study site (Season 1)	79	79	79
LMR study site (Season 2)	97	93	95
Gabon (Estuaire)	33	31	33
Gabon (Ogooue-Maritime)	57	56	57
Republic of Congo (Kouilou)	7	7	7
Republic of Congo (Lekoumou)	123	117	116
Republic of Congo (Likouala)	73	72	70
Republic of Congo (Pool)	71	69	71
**TOTAL**	**540**	**524**	**528**

**Table 2:** List of amphibian species sequenced in this study.

Family	Species	LMR	GO	RC	BINs	ABDG codes
**Order Gymnophiona**						
Dermophiidae	*Geotrypetes seraphini*			1	BOLD:ACM4617	85
**Order Anura**						
Arthroleptidae	*Arthroleptis adelphus*			1	BOLD:ACM4198	2
	"	7			BOLD:ACM4199	1
	*Arthroleptis perreti*	1			BOLD:ACM4527	4
	*Arthroleptis* cf. *poecilonotus*	2		9	BOLD:ACM4055	3
	*Arthroleptis sylvaticus*	2		10	BOLD:ACM4448	5
	*Arthroleptis taeniatus*	5			BOLD:ACM4581	8
	*Arthroleptis* sp. A	3			BOLD:ACM4312	7
	*Arthroleptis* sp. B			3	BOLD:ACM4462	6
	*Astylosternus batesi*			6	BOLD:ACM4639	53
	*Cardioglossa leucomystax*			3	BOLD:ACM4070	11
	*Cardioglossa gracilis*			1	BOLD:ACM4413	9
	*Cardioglossa gratiosa*			1	BOLD:ACM4021	10
	*Leptopelis aubryi*	8	5	3	BOLD:ACM4513 (1)	12
	*Leptopelis aubryioides*			2	BOLD:ACM4562	18
	*Leptopelis boulengeri*			1	BOLD:ACM4141	15
	*Leptopelis* sp. A			2	BOLD:ACM4288	16
	*Leptopelis brevirostris*	1			BOLD:ACM4142	13
	"			1	BOLD:ACM4258	14
	*Leptopelis* cf. *macrotis*			1	BOLD:ACM4257	19
	*Leptopelis ocellatus*			2	BOLD:ACM4119	17
	*Scotobleps gabonicus*			1	BOLD:ACM3974	73
Bufonidae	*Amietophrynus camerunensis*			19	BOLD:ACM4516 (3)	36
	*Amietophrynus funereus*			1	BOLD:ACM4379	39
	*Amietophrynus gracilipes*			18	BOLD:ACM4666	38
	*Amietophrynus gutturalis*			5	BOLD:ABX1848	37
	*Amietophrynus pusilla*			32	BOLD:ABX1889 (8)	42
	*Amietophrynus regularis*	1	5	12	BOLD:ACM4600	41
	*Amietophrynus tuberosus*			5	BOLD:ACM4608	40
	*Nectophryne afra*	2			BOLD:ACM4101	35
	"	1			BOLD:ACM4102	35
Conrauidae	*Conraua crassipes*			3	BOLD:ACM4265	78
Dicroglossidae	*Hoplobatrachus occipitalis*	2	4	9	BOLD:ABX2327	79
Hemisotidae	*Hemisus perreti*	3	1		BOLD:ACM4439	84
	*Hemisus guieensis*			4	BOLD:ACM4440	83
Hyperoliidae	*Afrixalus dorsalis*		7		BOLD:ACM4276	32
	"		1		BOLD:ACM4277	33
	*Afrixalus fulvovittatus*			10	BOLD:ACM3990	31
	*Afrixalus osorioi*			1	BOLD:ACM4091	30
	"			1	BOLD:ACM3992	29
	*Cryptothylax greshoffii*			6	BOLD:ACM4279	34
	*Hyperolius adspersus*	15	9		BOLD:ACM4236	20
	"			1	BOLD:ACM3931	20
	"		1		BOLD:ACM4235	20
	*Hyperolius olivaceus*		5		BOLD:ACM4149	22
	"			5	BOLD:ACM4150	22
	"	8	7		BOLD:ACM4237	22
	*Hyperolius dartevellei*			4	BOLD:ACM4221	21
	*Hyperolius ocellatus*	1			BOLD:ACM4613	24
	*Hyperolius pardalis*			1	BOLD:ACM4378	26
	*Hyperolius phantasticus*	5	7		BOLD:ACM4699	25
	*Hyperolius platyceps*		1		BOLD:ACM3935	23
	"	4			BOLD:ACM3936	23
	*Hyperolius tuberculatus*	3	10		BOLD:ACM4028	27
	*Phlyctimantis leonardi*		3		BOLD:ACM4536	28
	"		7	1	BOLD:ACY0609	28
Phrynobatrachidae	*Phrynobatrachus africanus*			2	BOLD:ACM4519	59
	"	1			BOLD:ACM4520	57
	"			1	BOLD:ACM4521	58
	"			1	BOLD:ACM4518	60
	*Phrynobatrachus auritus*	40			BOLD:ACM3966	54
	"			4	BOLD:ACM3967	54
	"			1	BOLD:ACM4483	55
	*Phrynobatrachus batesii*			1	BOLD:ACM3923	61
	*Phrynobatrachus* cf. *hylaios*			7	BOLD:ACM4501	66
	*Phrynobatrachus horsti*			1	BOLD:ACM3921	63
	*Phrynobatrachus ruthbeateae*			1	BOLD:ACM3922	62
	*Phrynobatrachus* sp. A	21	1		BOLD:ACM4053	56
	*Phrynobatrachus* sp. B			2	BOLD:ACM4606	65
	*Phrynobatrachus* sp. C			1	BOLD:ACM4544	64
Pipidae	*Hymenochirus curtipes*			5	BOLD:ACM4325	74
	*Hymenochirus* sp.			1	BOLD:ACM4340	75
	*Xenopus andrei*	1		2	BOLD:AAH9248	67
	*Xenopus pygmaeus*			8	BOLD:AAW7585	68
	*Xenopus boumbaensis*			1	BOLD:AAH7184	69
	*Xenopus epitropicalis*			3	BOLD:AAJ6803	70
	*Xenopus mellotropicalis*	7	4		BOLD:AAH0940	72
	"			7	BOLD:AAH0942	71
Ptychadenidae	*Ptychadena mascareniensis* 6*			7	BOLD:ACH6774	77
	*Ptychadena porosissima*	16	2		BOLD:ACM4178 (3)	43
	*Ptychadena taenioscelis*	6		3	BOLD:ACM4023 (2)	76
	*Ptychadena uzungwensis*			1	BOLD:ACM4112	44
	*Ptychadena* sp. A	4			BOLD:ACM4315	46
	*Ptychadena* sp. B			5	BOLD:ACM4111	47
	*Ptychadena* sp. C	3	1		BOLD:ACM4022	45
	"		4		BOLD:ACM4572	45
Pyxicephalidae	*Aubria masako*			3	BOLD:ACM3984	82
Ranidae	*Amnirana albolabris*	4			BOLD:ACM3918	48
	"			1	BOLD:ACM4391	50
	"			9	BOLD:ACM4408	49
	*Amnirana* cf. *amnicola*			3	BOLD:ACM4594	51
	*Amnirana lepus*			7	BOLD:ACM4529	52
Rhacophoridae	*Chiromantis rufescens*			1	BOLD:ACM4211	81
	"		5		BOLD:ACM4212 (1)	80

The number of specimens collected at the Loubomo-Mougagara Road study site are indicated in column LMR, those from other sites in Gabon in the GO column, and the number of specimens from the Republic of Congo in the RC column. A species name listed twice indicates that multiple BINs were assigned the same name.

The number in parentheses in the BINs column represents the number of specimens with no COI data. While these specimens were not assigned to a BIN, 16S data strongly suggest that they would be in that BIN.

* OTU 6; sensu Zimkus et al. 2016.

### Dermophiidae

#### *Geotrypetes seraphini* (Duméril, 1859)

We sequenced one specimen (USNM 576262) from Mayongongo, RC. The 16S sequence is a 99% match to a *G*. *seraphini* specimen collected in Gabon (FMNH 256782), used in both Frost et al. (DQ283337 [60]) and Roelants and Bossuyt (AY523754 [61]). Our 16S sequence is 97% similar to a *G*. *seraphini* specimen (BMNH 2005.2) from Cameroon [62]. Though our COI sequence was 94% similar to the BMNH specimen, it was placed in a separate COI BIN and ABDG group.

### Arthroleptidae (S1 Fig)

#### Arthroleptis

Six species of *Arthroleptis* are known to occur in Gabon [42, 63–65], although 10 different morphospecies were identified during fieldwork at the LMR site. Seven specimens (USNM 558505–11) identified at the LMR site were placed in one BIN and two ABDG groups, and one specimen (USNM 584017) from the RC was placed in another BIN and ABDG group. These specimens form a 16S clade with *A*. *adelphus* samples from GenBank, with LMR site specimens genetically close to one sample (FJ151055) from Bioko Island, Equatorial Guinea, and the RC sample sister to GenBank samples (FJ151081–82) from Cameroon. Other GenBank samples (FJ151092–93, FJ151141), also from Cameroon, were placed more basal to our two clades. The type locality for *A*. *adelphus* is near Sangmelima, Cameroon; therefore, we treat all of these specimens as *A*. *adelphus*. However, the geographic and genetic structure suggests this may represent a species complex in need of further investigation.

We initially identified one specimen (USNM 558516) from Gabon as *A*. *variabilis*. The COI sequence is a 93% match to GenBank AB612010 identified as *A*. *variabilis* (ZFMK 68794 [66]). This specimen is placed in a 16S clade with the same GenBank specimen for which COI data are available (16S-AB612012, ZFMK 68794), but also with other specimens including the holotype (MCZ A-137978) and three paratypes (MCZ A-136931–32, A137980) of *A*. *perreti* [67] in GenBank (FJ151138–39, FJ151094–95) that are 94.2–96.4% similar to our specimen based on 16S data. Several other specimens in GenBank identified as *A*. *variabilis* (e.g. EU350212–13, DQ283081, FJ151069–70) from Cameroon, Equatorial Guinea, and elsewhere [60, 68, 69] are sister to *A*. *palava*, and this clade is sister to our *A*. *perreti* clade. Some of these *A*. *variablilis* (FJ151069–70) were compared to type material of *A*. *variabilis* and matched the description [68]. Therefore, we treat our specimen USNM 558516 as *A*. *perreti*.

Two specimens (USNM 558512–13) recognized as an *Arthroleptis* species from the LMR study site in Gabon and nine (USNM 573378, USNM 584016, USNM 584018–19, USNM 584053–55, USNM 584405–06) from the RC all placed in one BIN and one ABDG group. Our specimens form a 16S clade with some specimens in GenBank identified as *A*. *poecilonotus* (e.g. FJ151084–85, FJ151089–90, FJ151140) from Cameroon. However, there are three 16S clades of *A*. *poecilonotus* among the GenBank samples. Blackburn [68] identified three clades of *A*. *poecilonotus*: "Cameroon, Togo Hills, and Western [Ghana + Sierra Leone]". The type locality for *A*. *poecilonotus* is Ghana [63]. Two names placed in synonymy with *A*. *poecilonotus* (*A*. *macrodactylus* and *A*. *inguinalis*) have type localities in Gabon [63]. One specimen from Blackburn [68] was labeled as *A*. *brevipes* in GenBank (FJ151107), but treated as "*A*. *poecilonotus*, Togo Hills" in the manuscript [68]. The type locality for *A*. *brevipes* is "Bismarckburg, Togo" [63]. Therefore, the Western [Ghana + Sierra Leone] clade might be treated as *A*. *poecilonotus* and the Togo Hills populations as *A*. *brevipes*. Likely, either *A*. *macrodactylus* or *A*. *inguinalis* is the name that applies to our specimens and the specimens in GenBank from Cameroon. We compared our specimens with the descriptions of both *A*. *macrodactylus* and *A*. *inguinalis*. The only major diagnostic feature for *A*. *macrodactylus* is possession of a third finger twice as long as the second, but this is based on a single juvenile specimen. Our specimens have elongate third fingers, but none are twice as long as the second finger. For now, we treat our specimens as *A*. cf. *poecilonotus*, which is consistent with the "*A*. *poecilonotus* Cameroon clade" of Blackburn [68].

We found three clades closely related to *A*. *sylvaticus* placed in three BINs and three ABDG groups. One clade consisted of two specimens (USNM 558514–15) from the LMR site in Gabon and 10 (USNM 565012, USNM 576272, USNM 584001–08) from the RC that were placed in a 16S clade with GenBank material, close to other sequences identified as *A*. *sylvaticus*, DQ283078 from Cameroon [60] and close to others from Cameroon (FJ151075, FJ151106, DQ022349). The type locality for *A*. *sylvaticus* is ‴Buta', Uele, Dem. Rep. of Congo" [63]. Specimens from the neighboring RC, in this wide-ranging clade, are the closest sampled to the type locality; thus, we refer to this clade as *A*. *sylvaticus*. The other two clades placed close to *A*. *sylvaticus* included three specimens (USNM 558517–19) from the LMR site placed in one COI BIN and ABDG group and three specimens (USNM 584059–61) from the RC placed in another COI BIN and ABDG group; the 16S data placed them stepwise sister to our *A*. *sylvaticus* clade. These two clades showed 15 and 17% uncorrected sequence divergence respectively for 16S, which included some from the same population as one of our *A*. *sylvaticus* specimens. Thus, we treat these as "*Arthroleptis* sp. A" (USNM 558517–19) and "*Arthroleptis* sp. B" (USNM 584059–61).

Four specimens initially identified as *"Arthroleptis* sp." (USNM 558521–24) from the LMR site in Gabon were placed in their own COI BIN and ABDG group and were in a 16S clade with specimens from GenBank labeled as *A*. *sylvaticus* (FJ151144) from southwestern Cameroon and (FJ151062) from Equatorial Guinea [68], and a specimen labeled as *A*. *taeniatus* (DQ283232) from Equatorial Guinea [60]. A single individual (USNM558520) was initially identified as *Cardioglossa*, but the barcode data identified it as *A*. *taeniatus* (see below). The type locality of *A*. *taeniatus* is "Zima" (= Sangmelima), South Cameroon (fide Frost 2016). Because the 12 other specimens from Gabon and the RC that we identified as *A*. *sylvaticus* (see above) were more distant genetically to these GenBank specimens (FJ151144, FJ151062) and our specimens (USNM 558520–24), we treat this clade as *A*. *taeniatus*.

#### Astylosternus

Of the twelve recognized species of *Astylosternus*, only a single species, *A*. *batesi*, is known to occur in Gabon [42, 63–65]. Although no individuals of this genus were identified at the LMR site, six specimens (USNM 584066–71) identified as *A*. *batesi* from RC were placed in a single BIN and ABDG group. These form a 16S clade with one *A*. *batesi* from GenBank (FJ151071), two specimens identified as *A*. *schioetzi* (AF124108, DQ283349, both from Cameroon) and one identified as *A*. *diadematus* in GenBank (AY341723), all with <2–5% sequence divergence; *A*. *batesi* and *A*. *schioetzi* were polyphyletic with respect to each other, and the *A*. *diadematus* sequence was placed most basal. The latter two species are not known to occur in the RC. Therefore, the identification of the GenBank specimens should be verified, as they may represent the more widespread *A*. *batesi*, which may be a multiple species complex, or represent one or two morphologically variable species.

#### Cardioglossa

Five species of *Cardioglossa* have been identified in Gabon [42, 63–65]. A single individual from the LMR site (USNM 558520) was initially identified as *Cardioglossa* (mentioned above), but barcode data placed it with *Arthroleptis taeniatus*, and secondary examination of the specimen confirmed this identification. Therefore, no *Cardioglossa* were observed at the LMR site in Gabon. We included three species of *Cardioglossa* from the RC: *C*. *gracilis* (USNM 584072), *C*. *gratiosa* (USNM 584074, COI only) and *C*. *leucomystax* (USNM 584075–76, USNM Herp Tissue 3), each placed in their own COI BINs and ABDG groups. The *C*. *gracilis 16S* sequences are nearly identical to other *C*. *gracilis* sequences in GenBank (EF640994, EF621773, FJ151065). Similarly, the *C*. *leucomystax 16S* sequences are nearly identical to other *C*. *leucomystax* sequences from GenBank (EF640991–93). Unfortunately, we were unable to obtain *16S* sequence data for our *C*. *gratiosa* sample.

#### Leptopelis

We identified eight *Leptopelis aubryi* (USNM 558525–29, USNM 580583–85) from the LMR site in Gabon, five specimens (USNM 578194–96, USNM 578240, USNM 578242) from the other sites in Gabon, and three specimens (USNM 584077–79) from the RC that were placed together in one BIN and ABDG group. The 16S data placed our *L*. *aubryi* in a clade with other samples of *L*. *aubryi* (KF888326–31) from Gabon and Cameroon [70].

We sequenced two *Leptopelis aubryioides* (USNM 584080–81) from the RC that were placed in their own COI BIN and ABDG group and in a 16S clade sister to a GenBank (KF888342) *L*. *millsoni* specimen from Cameroon, but with > 4.5% sequence divergence. We sequenced one *L*. *boulengeri* (USNM 584082) from the RC that was placed in its own COI BIN and ABDG group, and based on 16S data was placed with other *L*. *boulengeri* specimens in GenBank (KT967085–89) from Cameroon. Two *Leptopelis* (USNM 576026, USNM 576235), initially identified as *L*. *brevirostris* from the RC, were placed in their own COI BIN and ABDG group and based on 16S data were placed sister to a *Leptopelis bocagii* from Tanzania (GenBank DQ283418) with 6.5% sequence divergence from our samples. Morphologically, these specimens are different from *L*. *bocagii* or *L*. *brevirostris*; therefore, we treat them as *Leptopelis* sp. A. Two *Leptopelis ocellatus* (USNM 584083–84) from the RC were placed in their own COI BIN and ABDG group, and 16S data placed them sister to another large clade containing *L*. *aubryi* and *L*. *spiritusnoctis* samples. One *Leptopelis* cf. *notatus* (USNM 558530) from the LMR Gabon site and one *L*. *brevirostris* (USNM 576025) from the RC were placed in separate COI BINs and ABDG groups, and based on 16S data these specimens were placed sister to two specimens currently labeled as *L*. *brevirostris* in GenBank. One of these (AY702652) is now the type specimen of *L*. *crystallinoron* (ZFMK 73139 [71]). The other (AF215447) lacks voucher information. Because our specimens have conspicuous tympana, a character that is absent in *L*. *crystallinoron*, we treat ours as *L*. *brevirostris*.

One specimen identified only to *Leptopelis* sp. (USNM 584085) was placed in its own COI BIN and ABDG group, and was present in a 16S clade with *L*. *macrotis* samples (GenBank KF888338–41) from Guinea and Liberia, as well as one sample of *L*. *millsoni* (HQ130757) from the Democratic Republic of Congo [72], all within < 2.5% uncorrected pair-wise differences based on 572 bp of 16S data. The known distribution of *L*. *macrotis* ranges from Sierra Leone to Ghana, and our specimen USNM 584085 from the RC would extend the known range of *L*. *macrotis* considerably. Therefore, we tentatively refer to our specimen (USNM 584085) as "*L*. cf. *macrotis*" until further data become available and specimens can be compared.

#### Scotobleps

*Scotobleps* is a wide-ranging monotypic genus that occurs across Gabon. We did not observe any at the LMR site, but one specimen of *Scotobleps gabonicus* (USNM 584086) from the RC was sequenced. This specimen is in a 16S clade with three *S*. *gabonicus* in GenBank, one with unknown locality (AF215341), one (DQ283367) from Cameroon, and one (KC152645) from Equatorial Guinea, with 5–8% sequence divergence indicating substantial genetic variation within the limited geographic distribution of the taxon.

### Bufonidae (S2 Fig)

#### *Amietophrynus* (recently recognized as *Sclerophrys* [73])

At least eight species of *Amietophrynus* occur in Gabon [42, 63–65], but only *A*. *regularis* was observed at the LMR site. We sequenced one *Amietophrynus regularis* (USNM 580562) from the LMR site, five others (USNM 578227–29, USNM 578232, USNM 578254) from elsewhere in Gabon, and 12 (USNM 576083–85, USNM 576087–89, USNM 576396, USNM 576494, USNM 576534–37) from the RC. All were placed in one COI BIN and ABDG group and in a 16S clade with many other specimens ranging across West Africa and throughout the Congo Basin in GenBank identified as *A*. *regularis*. These samples had about 2% sequence divergence, indicating a widespread species with low amounts of genetic variation. Other "*A*. *regularis"* sequences in GenBank, placed in other species clades, are likely misidentifications.

We also sequenced 19 *Amietophrynus camerunensis* (USNM 576080–81, USNM 576395, USNM 576400–01, USNM 584091–98, USNM 584100, USNM 584129, USNM 584130, USNM 584132–33, USNM Herp Tissue 4) from the RC, all of which were placed in one COI BIN and ABDG group and in a 16S clade with other *A*. *camerunensis* specimens from GenBank. Eighteen *A*. *gracilipes* (USNM 576236–38, USNM 576240–43, USNM 576416–24, USNM 576428–29), five *Amietophrynus gutturalis* (USNM 576499–01, USNM 576527–28), 32 *Amietophrynus maculatus* (USNM 576393, USNM 576431–33, USNM 576435–46, USNM 584142–44, USNM 584146–57, USNM 584411) and five *Amietophrynus tuberosus* (USNM 584135–39) from the RC were all placed in conspecific COI BINs and ABDG groups, and were placed in conspecific 16S clades with samples from GenBank. The *A*. *maculatus* group has recently been revised [74] and our specimens are now referred to as *A*. *pusilla*. One *Amietophrynus funereus* (USNM 584134) from the RC was placed in a 16S clade with GenBank samples labeled *A*. *villiersi* and *A*. cf. *gracilipes*, and was different from our *A*. *gracilipes* and other *A*. *gracilipes* from GenBank. No other *A*. *funereus* sequences are currently available in GenBank. Further work is needed to verify the identifications of these specimens and this potential species complex; thus, we maintain the identification of our specimen as *A*. *funereus*.

#### Nectophryne

At least two species of *Nectophryne* occur in Gabon, *N*. *batesii* and *N*. *afra*, both of which have sequence data accessioned in GenBank [42, 63–65]. We collected three *Nectophryne* (USNM 558531–33) from the LMR site in Gabon, two initially identified as *N*. *afra*, and the third as *N*. *batesii*. These specimens were placed in two COI BINs and one ABDG group. Our specimens were placed with other *N*. *afra* from GenBank, creating a monophyletic clade of *N*. *afra* that was sister to GenBank sequences of *N*. *batesii*. Because our three specimens, all collected from the same locality, were placed in two COI BINs, and the one specimen (USNM 558533) was initially identified as a different species based on morphology, the one specimen might represent a cryptic species. For now we treat them all as *N*. *afra*, but note this could be a species complex in need of revision.

### Conrauidae (S3 Fig)

#### Conraua

*Conraua crassipes* is the only species of *Conraua* known to occur in Gabon, although we did not observe it at the LMR field site. We did sequence three individuals (USNM 584162–63, USNM Herp Tissue 5) from the RC that were placed in one COI BIN and ABDG group. Comparison of 16S sequence data from two individuals (USNM 584162–63) with GenBank sequences (DQ019600, DQ022355, DQ347305) confirmed their identification as *C*. *crassipes*.

### Dicroglossidae (S4 Fig)

#### Hoplobatrachus

We collected and sequenced two *Hoplobatrachus occipitalis* (USNM 580614–15) at the LMR site. Additionally, we sequenced four other individuals (USNM 578221–22, USNM 578226, USNM 578250) from elsewhere in Gabon and nine (USNM 576585–88, USNM 576591–93, USNM 584158, USNM–Herp Image 2845) from the RC. They are all very similar genetically and were placed in one COI BIN and ABDG group, and these sequences formed a 16S clade with other *H*. *occipitalis* sequences in GenBank (AB272599–600, AF261263, AY014373–74, DQ283059, DQ347291, EU979845–46, GQ183571, KF991268; note *H*. *occipitalis* AY341689 and DQ346979 do not share overlapping 16S data in GenBank).

### Hemisotidae (S3 Fig)

#### Hemisus

*Hemisus perreti* has been previously recorded in coastal southwestern Gabon [42, 63–65]. We found three specimens of *Hemisus* (USNM 580586–88) at the LMR site, and additionally sequenced one individual (USNM 578247) from Ogooue-Maritime Province in Gabon and four individuals (USNM 576596–98, USNM Herp Tissue 6) from the RC. The Gabon and RC samples were placed in separate COI BINs and ABDG groups. These formed two 16S clades (corresponding to the two COI BINs and ABDG groups) that were step-wise sister to a clade of *H*. *marmoratus* samples from Tanzania (DQ283430, AY531831, AY326070) and Kenya (AY948749, AY364372). The Gabon samples were identified as *H*. *perreti* and sister to *H*. *marmoratus*, and the RC samples were sister to the *H*. *perreti* + *H*. *marmoratus* clade. The RC specimens (USNM 576596–98, USNM Herp Tissue 6) were identified as *H*. *guineensis* using the description by Frétey et al. [19]. The other samples from GenBank labeled as *H*. *marmoratus* and *H*. *sudanensis* (AF215342, AB612016, AB777217, KM509138 and AY531830, KC180076 respectively) were in a separate clade, sister to the (*H*. *guineensis* (*H*. *perreti + H*. *marmoratus*)) clade. We note that *H*. *sudanensis* currently is considered a synonym of *H*. *marmoratus* [63], but likely represents a distinct species based on our 16S tree. Further research is necessary to determine species boundaries within *H*. *marmoratus*.

### Hyperoliidae (S5 Fig)

#### Afrixalus

Five species of *Afrixalus* are thought to occur in Gabon [42, 63–65]: *A*. *dorsalis*, *A*. *laevis*, *A*. *paradorsalis*, *A*. *quadrivittatus*, *and A*. *fulvovittatus*. Eight specimens of *A*. *dorsalis* from the two other Gabonese sites were included. Seven (USNM 578099–100, 578103–04, 578106–08) from Estuaire and one (USNM 578102) from Ogooue-Maritime were placed in two COI BINs and ABDG groups (one for each province) and in one 16S clade. Only one record in GenBank for 16S is labeled as "*Afrixalus dorsalis"* (DQ347296); this specimen was collected from Bioko Island, Equatorial Guinea, and has been re-identified as *A*. *paradorsalis* at the host institution (CAS 207523) and is 97–98% similar to two other individuals in GenBank labeled *A*. *paradorsalis* (FJ151068, KM509083) from Cameroon; thus, CAS 207523 is likely to be *A*. *paradorsalis*. Our specimens form a single clade that may represent one or two species within *A*. *dorsalis*.

We sequenced 10 individuals of *Afrixalus fulvovittatus* (USNM 573412, USNM 573414–15, USNM 576600–03, USNM 576605–07) from the RC that were placed in one COI BIN and ABDG group. Our specimens formed a 16S clade with a specimen from GenBank (KF178889) identified as *A*. *quadrivittatus* from Gabon [75]; thus, these are likely part of the *A*. *"quadrivittatus–fulvovittatus"* species complex (see comments for both species in Frost 2016). We currently consider these to be *Afrixalus fulvovittatus*. We sequenced two other individuals identified as *Afrixalus osorioi* (USNM 565013, USNM 576608) from different localities in the RC that were placed in separate COI BINs and ABDG groups. Our specimens of *A*. *osorioi* formed a clade distinct from other *Afrixalus* 16S sequences in GenBank; no other *A*. *osorioi* were available in GenBank.

#### Cryptothylax

Of two known species of *Cryptothylax*, only *C*. *greshoffi* has been reported from Gabon [42, 63–65]. No individuals were found at the LMR site. However, we sequenced six individuals (USNM 576093, USNM 576095, USNM 576609–12) from the RC that were placed in one COI BIN and ABDG group. There are only two records of *C*. *greshoffi* in GenBank for 16S (AF215432, DQ283170). Our sequences form a clade with the two from GenBank, suggesting the current records support *C*. *greshoffi* as a single species.

#### Hyperolius

*Hyperolius* is a species-rich genus, with over 140 species recognized from throughout Sub-Saharan Africa [63], many of which represent species complexes in need of revision [76]. At least 12 species are known to occur in Gabon [42, 63–65]. We analyzed 15 specimens identified as *Hyperolius adspersus* (USNM 580591–93, USNM 580601, USNM 580611, USNM 558534–43) from the LMR site. We sequenced 10 additional samples (USNM Herp Image 2853, USNM 578163, USNM 578140, USNM 578142–43, USNM 578154, USNM 578156–58, USNM 578165) from other sites in Gabon and one (USNM 573365) from the RC. Most of our specimens were placed in a single COI BIN and form a 16S clade with other Gabonese sequences in GenBank (JQ863692–93, JQ863695–97); we note three of our specimens already have 16S data in GenBank, including USNM 578157 (JQ863693), USNM 578165 (JQ863694), and USNM 578142 (JQ863697) from Channing et al. [76]. Two of our specimens, one (USNM 573365) from the RC and one (USNM 578165) from Estuaire, Gabon, were each placed in their own COI BINs but all formed a single ABDG group. The 16S sequences were placed with one of our specimens in GenBank (JQ863694) and this clade was sister to the rest of our *H*. *adspersus* clade. A specimen from GenBank (KF178890) labeled as "*H*. cf. *adspersus*" from Gabon [75] is placed sister to our entire *H*. *adspersus* clade. Two additional specimens in GenBank, labeled as *H*. *adsperus* (JQ863714–15) from Angola are in a clade with other individuals in GenBank labeled as *H*. *dartevellei* and may be misidentified.

We sequenced eight individuals of *Hyperolius cinnamomeoventris* (USNM 558544–46, USNM 580602–03, USNM 580608–09, USNM 580590) from the LMR site and 12 from other Gabon sites, including seven (USNM 578115–17, USNM 578120–21, USNM 578136–37) from Ogooue-Maritime and five (USNM 578128–29, USNM 578131, USNM 578134, and USNM 578138) from Estuaire, and five others (USNM 584159–61, USNM 584416–17) from the RC. These sequences were all placed in three COI BINs: the first including all specimens from the LMR site and seven others from Ogooue-Maritime Gabon, the second with the five individuals from Estuaire, Gabon, and the third consisted of specimens from the RC yet all were placed in a single ABDG group. Our specimens form a single 16S clade with other individuals from GenBank (FJ594077, KP137115–17). This group was recently revised [77] and now our specimens are referred to as *H*. *olivaceus*. We note that the remainder of *Hyperolius cinnamomeoventris* is not monophyletic when all records in GenBank are included in our tree. Two specimens of *H*. *veithi* (GU443975, GU443988) are nested among some specimens of *H*. *cinnamomeoventris* (GU443989–90, HM064461; but see [77]) and one specimen of *H*. *phantasticus* (FJ594089; probably misidentified) is nested among other *H*. *cinnamomeoventris*. This species complex may still be in need of further revision [77].

We sequenced four individuals of *Hyperolius dartevellei* (USNM 576167–70) from the RC that were previously sequenced for 16S and deposited in GenBank [76]. The Congolese individuals formed one COI BIN and ABDG group and were placed in a 16S clade with other *H*. *dartvellei* specimens in GenBank. Three of these specimens were each listed as "USNM 576167" (JQ863653–55) in GenBank (presumably in error), of which our specimen USNM 576167 is identical to the sequence JQ863653, while our USNM 576168 is identical to the JQ863654–5 sequences. The fourth specimen, JQ863656 is listed as USNM 576170 in GenBank and is identical our USNM 576170. There are two clades of *H*. *dartevellei* in our 16S alignment including samples from GenBank, and these clades do not form a monophyletic clade as specimens identified as *H*. *adspersus*, *H*. *nasutus* and *H*. *rwandae* are nested among them. This result may be an artifact of the small amount of 16S sequence data used in our study compared to others [76]. One clade represents material from Zambia and Zimbabwe (with *H*. *rwandae*), while the other represents material from the Congo River Basin (with *H*. *nasutus*). Our samples were present in the clade with other individuals from the Congo River Basin, which is closer to the type locality of *H*. *dartevellei* [76].

We sequenced one individual of *Hyperolius ocellatus* (USNM 558547) from the LMR site in Gabon. This specimen is in a clade with three other individuals identified as *H*. *ocellatus* in GenBank (FJ594087, JX564872, KF693379) from Gabon and Cameroon. We note a fourth sequence in GenBank identified as *H*. *ocellatus* (AY603988) from Equatorial Guinea that is likely a different species. This sequence is placed with another specimen identified as *H*. *mosaicus* and is sister to a group that contains *H*. *jackie*, *H*. *lateralis*, *H*. *discodactylus*, *H*. *constellatus*, and *H*. *castaneus*. This specimen (CAS 207321) is currently identified as "*H*. cf. *ocellatus"* at its host institution and likely represents a different species (e.g. *H*. *mosaicus*).

We sequenced one individual of *Hyperolius pardalis* (USNM 573366) from the RC. This individual is sister to a specimen in GenBank identified as *H*. *pardalis* (AY323922), and these two are sister to our clade of *H*. *phantasticus*.

We sequenced five individuals of *Hyperolius phantasticus* (USNM 580606, USNM 558548–51) from the LMR site and seven others (USNM 578119, USNM 578167–68, USNM 578172, USNM 578174, USNM 578181, USNM Herp Image 2852) from Ogooue-Maritime in Gabon that were all placed in one COI BIN and ABDG group. Two sequences of 16S identified as *Hyperolius phantasticus* from Gabon are in GenBank. One sequence (FJ594088) is identical to a specimen identified as *H*. *glandicolor* from Kenya (FJ594081), and the other (FJ594089) is 99% similar to several *H*. *cinnamomeoventris*, and nested in one of the *H*. *cinnamomeoventris* clades (mentioned above). A third specimen identified as "*H*. sp. C *Phantasticus*" in GenBank (FJ594099) also from Gabon is placed with our *H*. *phantasticus* specimens in a clade that is sister to *H*. *pardalis* (see above). All three *H*. "*phantasticus*" in GenBank were included in a study by Veith et al. [78]. Two of these *Hyperolius* sequences of Veith et al. [78] have the same voucher number (RABI 150); however, one specimen (FJ594088) listed from "Rabi, Gabon," (identified as *H*. *phantasticus*) is genetically very different from the specimen identified as "*H*. sp. C *Phantasticus*" (FJ594099) that was collected from “Rabi oilfields, Gabon.” These specimens are listed as field numbers and do not appear to be vouchered at an institution. Because of the problems associated with these "*H*. *phantasticus"* specimens, one being identical to *H*. *glandicolor* (FJ594088), one nearly identical to *H*. *cinnamomeoventris* (FJ594089), and the duplicate voucher for FJ594088 and FJ594099 (RABI 150), the true identity of these specimens cannot be verified. Thus, we maintain our samples as *H*. *phantasticus*.

We sequenced four specimens of *Hyperolius platyceps* (USNM 558552–55) from the LMR site and one (USNM 578182) from Ogooue-Maritime Gabon that were put in two separate COI BINs and one ABDG group. These individuals form a 16S clade containing one individual from GenBank identified as *H*. cf. *platyceps* (FJ594091); no other *H*. *platyceps* sequences are present in GenBank.

We sequenced three individuals identified as *Hyperolius tuberculatus* (USNM 558556–58) from the LMR site and 10 (USNM 578122–23, USNM 578126–27, USNM 578183–88) from elsewhere in Gabon; all sequences were placed in one COI BIN and ABDG group. These individuals form a 16S clade with two individuals from GenBank identified as *H*. *tuberculatus* (AY323921, FJ594095); one of them represents the "*H*. *tuberculatus* B" (FJ594095) clade of Veith et al. [78]. The only other individual in GenBank (FJ594094) identified as *H*. *tuberculatus* is the "*H*. *tuberculatus* A" clade of Veith et al. [78]. These GenBank sequences (FJ594094–95) are from specimens collected in Mt. Cristal and Mt. Doudou (both in Gabon), and the type locality (Lambaréné) lies between them. Careful examination of these specimens and comparisons to the type description are needed to determine which, if either, represents the true *H*. *tuberculatus*.

#### Phlyctimantis

Of the four species belonging to the genus *Phlyctimantis*, *P*. *boulengeri and P*. *leonardi* are known to occur in Gabon [42, 63–65]. Although many individuals, presumably *P*. *leonardi* given its previous documentation in nearby areas [65], were heard during LMR field sampling, we were unable to collect any specimens from the LMR site. However, we did include 10 *Phlyctimantis* specimens from other Gabon sites and one from the RC that were placed in two COI BINs: three specimens (USNM 578202–03, USNM 578246) from Estuaire, Gabon were placed in one BIN, while seven (USNM 578197–201, USNM Herp Tissue 9, USNM 578238) from Ogooue-Maritime, Gabon and the single specimen (USNM 558742) from the RC were placed in a second BIN. All specimens were placed in a single ABDG group. There is currently a single 16S record in GenBank identified as *P*. *leonardi* (DQ283356). Our specimens form a clade with this GenBank record. For this reason, we refer to our specimens as *P*. *leonardi*.

### Phrynobatrachidae (S6 Fig)

#### Phrynobatrachus

We collected one *Phrynobatrachus africanus* (USNM 558581) at the LMR site in Gabon and sequenced four (USNM 584170–72, USNM Herp Tissue 8) from the RC. These sequences were placed in four COI BINs and ABDG groups. Our sequences placed with many other 16S sequences from GenBank (DQ022356, DQ283175, DQ347319, FJ769116–22, GU457530–31, KF693382) labeled as *P*. *africanus* from throughout the range of this species in west Central Africa. This clade shows significant structure associated with long branches and likely represents a species complex in need of further taxonomic work. Our Gabonese sample is genetically most similar to DQ283175, which is from Loango National Park in Gabon [60]. Our samples from the RC are scattered elsewhere in this clade. We assign all of our samples to *Phrynobatrachus africanus* until this complex is further analyzed.

Forty specimens (USNM Herp Tissue 1–2, USNM 558559–78, USNM 580565, USNM 580573, USNM 580576, USNM 580579–80, USNM 580619, USNM 580621, USNM 580627–28, USNM 580631, USNM 580635, USNM 580638, USNM 580642–44, USNM 580661, USNM 580663–64) from the LMR site, 25 of which were initially identified as *P*. *auritus*, were placed in one COI BIN. Four individuals (USNM 584165–68) from the RC were placed in another COI BIN, and formed a single ABDG group with the other forty *P*. *auritus*. One individual (USNM 563707) was placed in its own COI BIN and ABDG group. All of these were placed in a 16S clade with a single *P*. *auritus* GenBank specimen (FJ889454) from Uganda [79].

The previously mentioned *P*. *auritus* clade is sister to another clade containing 21 other specimens (USNM 558579–80, USNM 580564, USNM 580566–67, USNM 580570–72, USNM 580574–75, USNM 580578, USNM 580618, USNM 580620, USNM 580622, USNM 580629–30, USNM 580634, USNM 580636, USNM 580639, USNM 580640, USNM 580660) from the LMR Gabon site, and one specimen (USNM 578213) from Ogooue-Maritime, Gabon. The LMR specimens were initially grouped into ten different morphospecies, some of which were initially thought to be *Arthroleptis*. Although these specimens were collected at some of the same localities as the previously identified *P*. *auritus* clade, this group of specimens was placed in one COI BIN and ABDG group that was different from the *P*. *auritus* samples above. Thus, *P*. *auritus* from Gabon appears to be a species complex and in need of revision. Accordingly, we refer to the latter clade specimens as "*Phrynobatrachus* sp. A". Another clade including specimens from GenBank identified as *P*. *auritus* (DQ022362, DQ283084, FJ769123–25, FJ889454, KJ626406–11) contains individuals from Equatorial Guinea and Cameroon. The type locality of *P*. *auritus* is "Benito River, north of the Gaboon River between 20 and 30 miles inland from the coast, Gaboon (Gabon)" (fide Frost 2016). Our analyses point to the need for a detailed comparison of these and additional specimens, including type material, to determine which clade is the true *P*. *auritus*.

We sequenced one specimen of *Phrynobatrachus batesii* (USNM 584173) from the RC; it was placed in its own COI BIN and ABDG group and clusters with other specimens of *Phrynobatrachus batesii* from GenBank (EU718715, FJ769113–15, JQ711171–72).

We sequenced seven specimens initially identified as *Phrynobatrachus hylaios* (USNM 565017–18, USNM 573418, USNM 576676–77, USNM 576680–81) from the RC. These specimens cluster with two sequences from GenBank (GU457542–43) initially identified as "*P*. cf. *hylaios*," but are likely part of the *Phrynobatrachus latifrons* complex. Therefore, we recognize our specimens as "*P*. cf. *hylaios*" until further work is done on this group.

We sequenced two *Phrynobatrachus ruthbeateae* (USNM 584174, USNM Herp Tissue 7) from different localities in the RC that were each placed in their own COI BINs and ABDG groups. These were placed in a 16S clade with other *P*. *ruthbeateae* from GenBank (JQ711167–70, KF020537–38). However, recently Rödel et al. [80] described a new species, *Phyrnobatrachus horsti*, to which they referred MCZ A-147865 and 147891 (sequences KF020537–38, previously considered and currently labeled as *P*. *ruthbeateae* in GenBank). Our sample, USNM Herp Tissue 7, is sister to these two and matches the description of this new species; therefore, we refer it to *P*. *horsti*.

We also sequenced two specimens of "*Phrynobatrachus* sp." (USNM 573420–21) from the RC, which were placed in one COI BIN and ABDG group, and are sister to specimens in GenBank identified as *P*. *francisci* (AY902377, EU718720, GU457546–49). *Phrynobatrachus francisci* is not known to occur in the RC; these specimens do not match the description of *P*. *francisci*, and our sequences have 7–8% sequence divergence from the GenBank sequences. Therefore, we refer to our specimens (USNM 573420–21) as "*Phrynobatrachus* sp. B."

We sequenced one specimen of *Phrynobatrachus* that initially was identified as *P*. *cornutus* (USNM 584164) from the RC and placed in its own COI BIN and ABDG group. Its 16S sequence is unique and placed it sister to specimens in GenBank identified as *P*. *dispar*, *P*. *leveleve* and *P*. *mababiensis*. This specimen is morphologically similar to those called *P*. *ogoensis*, though our specimen lacks webbing on the feet, a trait characteristic of *P*. *ogoensis*. The *P*. *ogoensis* specimen in GenBank (KP247505, MCZ A–149217 [81]) was recently re-identified as the newly described *P*. *mayokoensis* [80], which our 16S tree supports. No other name appears to be available for our specimen, and it is more than 4% sequence divergent from the *P*. *mayokoensis* 16S sequences in GenBank (KR827545–46). Thus, we treat USNM 584164 as "*Phrynobatrachus* sp. C."

### Pipidae (S7 Fig)

#### Hymenochirus

Of the four named species of *Hymenochirus*, at least two are documented from Gabon [42, 63–65]. Although no *Hymenochirus* individuals were found at the LMR site in Gabon, we sequenced six individuals from the RC, five of which (USNM 563881, USNM 576617–20) were placed in one COI BIN and ABDG group and morphologically identified as *Hymenochirus curtipes*. Another specimen (USNM 584175) was placed in a separate COI BIN and ABDG group and identified only as *Hymenochirus* sp. The only 16S data available from GenBank are *H*. *boettgeri* (AY341726, AY523756) and "*H*. sp. BJE 2004" (AY581623; country unknown). Our barcode results support our identification that *H*. *curtipes* and the unidentified species (*H*. sp. BJE 2004) appear to be different species. Our two species are placed sister to one another, and they are sister to an *H*. *boettgeri* + "*H*. sp. BJE 2004" clade based on 16S data. *Hymenochirus feae* and *H*. *boulengeri* have reported distributions from Gabon and Northeastern DRC, respectively [63]. Our specimen was examined and compared with the Frétey et al. [19] key but we could not identify it to species. Based solely on these known distributions and the locality of the *Hymenochirus* sp. sampled, it is more likely that this specimen is *H*. *feae* or an undescribed species.

#### Xenopus

Five species of *Xenopus* are reported in Gabon [42, 63–65, 82], and two were identified at the LMR site. We sequenced six specimens (USNM 580552–55, USNM 580557–58) initially identified as *Xenopus epitropicalis* from the LMR site in Gabon, four specimens (USNM 578205–08) from elsewhere in Gabon, and 10 specimens (USNM 573442–48, USNM 576613–14, USNM 584434) from the RC. We compared our sequence data to those published in GenBank for which 16S data were available, including those of a recent study that has identified six new polyploid species of *Xenopus* [82]. All of our specimens initially identified as *X*. *epitropicalis* from Gabon were placed in one COI BIN and ABDG group, and 16S data placed them with *X*. *mellotropicalis* based on the 16S data from Evans et al. [82] and other samples in GenBank (AY581660–4). The *X*. *epitropicalis* from the RC were placed in two separate COI BINs and ABDG groups: the first containing seven individuals (USNM 573445–48, USNM 576613–14, USNM 584434) that were within a 16S clade that included *X*. *mellotropicalis*, and the second with three individuals (USNM 573442–44) that were within a 16S clade that included *X*. *epitropicalis*.

We initially identified one specimen (USNM 558582) from the LMR Gabon site as *Xenopus fraseri* and sequenced 11 others (USNM 576244–47, USNM 576250–51, USNM 584177–81) from the RC, all of which were placed in three COI BINs and ABDG groups. One individual (USNM576247) from the RC was placed in its own BIN and ABDG group, and the corresponding 16S sequence data was nearly identical to *X*. *boumbaensis* GenBank sequences. The remaining specimens were placed in two other BINs and ABDG groups. One group (USNM 558582, USNM 576250–51), including the specimen from the LMR initially identified as *X*. *fraseri*, was 99.6–99.8% identical to *X*. *andrei* (AY581627). The other group (USNM 576244–46, USNM 584177–81) was placed with specimens at base of the *X*. *allofraseri* clade with *X*. *pygmaeus* specimens (AY581626, HQ225690–1, JQ302191), rendering *X*. *pygmaeus* paraphyletic. Additional sampling across the Congo Basin, more molecular data, and more rigorous analyses may resolve *X*. *pygmaeus* as monophyletic. For now, we refer to these specimens from the RC as *X*. *pygmaeus*.

### Ptychadenidae (S8 Fig)

#### Ptychadena

*Ptychadena* is a species-rich genus with over 50 recognized species, many of which are known to include undescribed species (see [63]). Five species have been reported in Gabon: *P*. *aequiplicata*, *P*. *mascareniensis*, *P*. *perreti*, *P*. *pumilio* and *P*. *taenioscelis* [42, 63–65]. We collected 29 specimens from the LMR study site in Gabon initially identified as eight different morphospecies. We included an additional seven specimens from other localities in Gabon, and 16 individuals from the RC. Our COI data placed 16 specimens (USNM 558588–94, USNM 580646–51, USNM 580653–55) from the LMR site and two (USNM 578214, USNM 578248) from Ogooue-Maritime, Gabon in one BIN and ABDG group, and these sequences were placed in a 16S clade with *Ptychadena porosissima* specimens in GenBank (AF215411, DQ525941, KF027212). We note that 16S sequences for the specimen “AC2122” appears in GenBank twice (AY517601 [83] and DQ525941 [84], respectively) and the two sequences differ by 35 bp of 535 bp (6.5%). Until the correct sequence is associated with this specimen, neither can be trusted, but the DQ525941 sequence was placed in a clade with our specimens and other *P*. *porosissima* specimens in GenBank. Three specimens (USNM 580656–58) from our LMR site and five (USNM 578215–18, USNM 578239) from elsewhere in Gabon were placed in two COI BINS and one ABDG group and form a 16S clade with the aforementioned specimen identified as "*P*. *porosissima*" in GenBank (AY517601, AC2122). Therefore, we refer to these specimens as *Ptychadena* sp. C.

We sequenced five specimens (USNM 558583–87) from the LMR site that we initially identified as *Ptychadena pumilio*. These were placed in one COI BIN and ABDG group with a Gabonese specimen (USNM 580659) and three (USNM 576100, USNM 576692–93) from the RC identified only as *Ptychadena* sp. These sequences form a 16S clade with three individuals from GenBank (DQ525943, DQ071575, KF178893) identified as *P*. *taenioscelis*. Two other sequences in GenBank (AY517600, DQ525942) identified as *P*. *pumilio* are step-wise more distantly related to our clade. The latter more distantly related specimen (MOR Gu 212) is from "Guinea, Mt Bero," closer to the type locality of *P*. *pumilio* (Medine, Senegal), and reported by the same authors [83] as one of the specimens (DQ525943) in our clade identified as *P*. *taenioscelis*. *Ptychadena taenioscelis* was previously recognized as a subspecies of *P*. *pumilio*. Our data suggest that "*P*. *pumilio*" is still a species complex in need of further revision, and pending further study we identify our specimens as *Ptychadena taenioscelis*.

We sequenced 25 other individuals identified as either *Ptychadena perreti*, *P*. *mascareniensis*, or *P*. *aequiplicata;* seven (USNM 558595–97, USNM 580645, USNM 580656–58) were from our LMR site, five (USNM 578215–18, USNM 578239) from elsewhere in Gabon, and 13 (USNM 576099, USNM 576691, USNM 576694, USNM 576697–700, USNM 584195, USNM 584197–200, USNM 584203) from the RC. Four specimens (USNM 558595–97, USNM 580645) from the LMR site were placed in one COI BIN and ABDG group and form a 16S clade with one individual in GenBank (DQ525919) identified as "*P*. aff. *aequiplicata*" However, another clade of GenBank samples (AY517613–18, KF991275) identified as *P*. *aequiplicata*, is more distantly related to our four specimens, which are more closely related to specimens identified as *P*. *bibroni* (AY517602–3) and *P*. *perreti* (GQ183595, labeled *P*. *chrstyi*, see below). Our four specimens (USNM 558595–97, USNM 580645) are referred to as "*Ptychadena* sp. A," but we note that they are related to *P*. aff. *aequiplicata* of Vences et al. [84].

Five of the RC specimens (USNM 576099, USNM 584197–200) were initially identified as *P*. *perreti*, which appears to be another species complex. These specimens were placed in one COI BIN and ABDG group and form a 16S clade with one specimen in GenBank (GQ183595) initially identified as *Ptychadena christyi* (SL507) and another as *P*. aff. *bibroni* (AY517604 [84]). However, one of us (BMZ) re-identified the *P*. *christyi* specimen as *P*. *perreti* via preliminary molecular analyses of the genus. We note also that a juvenile specimen in GenBank (MCZ Herp: A-147907; KF178892) initially identified as *P*. *perreti* was been re-examined by one of us (BMZ) and re-identified as "*P*. sp. BMZ-2014a" because its taxonomic identity could not been confirmed. Because this complex still needs additional work, we refer to our specimens (USNM 576099, USNM 584197–200) in this complex as "*Ptychadena* sp. B."

*Ptychadena* "*mascareniensis*" is also recognized as a multispecies complex [63, 84, 85]. We sequenced seven individuals (USNM 576691, USNM 576694, USNM 576697–700, USNM 584202) from the RC originally identified as *Ptychadena* "*mascareniensis*" that were placed in one COI BIN and ABDG group and formed a 16S clade with specimens from GenBank identified by Measey et al. [83] as "*Ptychadena mascareniensis D*" (DQ525931). Although formerly only known from Kenya (see [63]), our results support the recent work by Zimkus et al. [85] who identified this clade (their OTU 6) across the Congo basin and as far west as Cameroon.

We sequenced one individual (USNM 584195) from the RC that forms a clade with several other specimens in GenBank identified as *Ptychadena uzungwensis* (DQ525945, KF178894, KF410853–54). Therefore, we refer to this specimen as *Ptychadena uzungwensis*.

### Pyxicephalidae (S9 Fig)

#### Aubria

Only two species of *Aubria*, *A*. *masako* and *A*. *subsigillata*, are currently recognized and both occur in Gabon [42, 63–65], although none were encountered at the LMR site. We sequenced three individuals of *A*. *masako* (USNM 576048, USNM 576105–06) from RC, and they were placed in one COI BIN and ABDG group. Sequence data of the *16S* gene are available for only four specimens of *A*. *subsigillata* in GenBank, and our specimens appear genetically distinct from these; the first three sequences (DQ283173, DQ283352, KF991276) are 12–13% divergent from the three RC specimens. The other GenBank sample (Y11975) identified as *A*. *subsigillata* is 24–26% divergent from our sample and the other samples in GenBank. We suspect that it is likely misidentified because when a blast search of the sequence is completed, the sequence matches more closely with sequences in the family Ranidae, and it is placed outside of our *Aubria* + *Chiromantis* 16S clade.

### Ranidae (S9 Fig)

#### Amnirana

We note several species in the genus *Hylarana* were recently removed from synonymy and placed in a resurrected *Amnirana* genus; thus, these specimens are still labeled "*Hylarana*" in GenBank records. Only three species of *Amnirana* have been reported from Gabon: *A*. *albolabris*, *A*. *amnicola* and *A*. *lepus* [42, 63–65]. A total of 14 specimens were identified as *A*. *albolabris*: four (USNM 558598–601) from the LMR site and 10 (USNM 558743, USNM 584204–11, USNM 584437) from the RC. Additionally, three individuals (USNM 584212–14) from the RC were identified as *A*. *amnicola*, and seven others (USNM 584215–21) from the RC were identified as *A*. *lepus*. The COI data placed our Gabonese *A*. *albolabris* in one BIN and ABDG group, the RC *A*. *albolabris* in two BINs and ABDG groups, and *A*. *amnicola* and *A*. *lepus* each in their own BIN and ABDG group. We included all species available in GenBank for 16S comparisons.

Our *A*. *albolabris* specimens form two 16S clades: one clade of specimens from Gabon and the other including specimens from the RC and GenBank sequences of Cameroonian specimens (DQ022351, KR264110, KR264122) identified as "*A*. *albolabris* 1" in Oliver et al. [86]. Additional GenBank specimens identified as "*A*. *albolabris*" were placed elsewhere in the tree. One sequence (JX564871) was placed with *A*. *galamensis*, another (DQ283369) was placed with *A*. *amnicola* (see below), and a third (KR264062) identified as "*A*. cf. *albolabris*" from Sierra Leone with two "*A*. *albolabris* 2" from Liberia (KR264106–7). These latter three GenBank sequences were from Oliver et al. [86]. The type locality for *A*. *albolabris* was reported as "West Africa" (Hallowell, 1856) and was later restricted to Gabon (Perret, 1977). We consider our clade to represent the true *A*. *albolabris*, because it contains specimens from Gabon and the RC that are closest to the type locality. This clade includes specimens identified as "*A*. *albolabris* 1" in Oliver et al. [86]. The other specimen from GenBank labeled "*A*. *albilabris"* from Cameroon (DQ283369) was placed with our *A*. *amnicola* specimens. The type locality for *A*. *amnicola* is "Ilanga, Eséka, Cameroun méridional" [63]. The *A*. *amnicola* specimens from GenBank (KR264034–36) are from Cameroon, East and Southwest provinces [86], and closer to the type locality; they form a clade sister to our *A*. *albolabris* + *A*. *amnicola* clade. Therefore, we refer to our specimens as "*A*. cf. *amnicola*," and note this clade may represent a new species. This complex is in need of revision.

Our seven *A*. *lepus* (USNM 584215–21) from the RC were placed in one COI BIN. One of our specimens (USNM 584220) of *A*. *lepus* from the RC was previously sequenced for 16S and deposited in GenBank (KR264112 [86]), but differs by two base-pairs from our sequence. All of our *A*. *lepus* were identical for 16S, and form a clade sister to three other *A*. *lepus* in GenBank from Cameroon (AY014377, KR264067–68).

### Rhacophoridae (S9 Fig)

#### Chiromanti

*Chiromantis rufescens* is the only species in the genus known to occur in Gabon [42, 63–65]; none were found at the LMR site. We sequenced five specimens (USNM 578109, USNM 578111–14) of this species from Estuaire, Gabon and an additional specimen (USNM 584222) from the RC. These were placed in separate COI BINs and ABDG groups, and all formed a 16S clade with additional sequences from GenBank (AF458126, AF215347, AY341721, AY880494, DQ347297, GQ204724, KF991282) from Equatorial Guinea, Cameroon, and elsewhere. They appear to represent a single species with some geographic variation.

### Comparing field identifications to DNA barcode identifications

While our original field identifications estimated 48 amphibian species belonging to 13 genera, barcoding identified 28 species from 11 genera among the LMR samples in both seasons (Table 3). Overall, fewer than 50% of specimens were correctly identified in the field on the basis of morphology alone. Here we define a ‘correct identification’ as an individual that was assigned a name in the field with the same taxonomic designation suggested by the post-hoc DNA barcode analysis, or, in the case of unresolved or new (since 2013) taxonomy, if a unique set of individuals were correctly assigned to a single morphospecies in the field that aligned with an operational taxonomic unit (OTU) following the DNA barcoding. On the other hand, if individual specimens within a single barcode cluster, or OTU, were identified in the field as multiple species, we considered all of those specimens to be ‘misidentified.’ Misidentifications were attributed to a lack of training and experience in character recognition, significant polymorphisms within a group, and/or likely cryptic diversity within a group.

**Table 3:** Number of species/morphospecies of each genus identified in the field versus identifications based on DNA barcode analyses for each sampling season and overall in the LMR study area in Gabon.

	Dry season		Wet season		Combined	
Genus	Field	Barcode	Field	Barcode	Field	Barcode
*Alexteroon*	-	-	1	0	1	0
*Amietophrynus*	1	1	-	-	1	1
*Amnirana*	-	-	1	1	1	1
*Arthroleptis*	8	0	5	6	10	6
*Cardioglossa*	1	0	-	-	1	0
*Hemisus*	1	1	-	-	1	1
*Hoplobatrachus*	1	1	-	-	1	1
*Hyperolius*	6	3	7	6	10	6
*Leptopelis*	1	1	2	2	2	2
*Nectophryne*	-	-	2	1	2	1
*Phrynobatrachus*	8	2	2	3	8	3
*Ptychadena*	7	4	3	3	8	4
*Xenopus*	1	1	1	1	2	2
Totals	35	14	24	23	48	28

The frogs that were most difficult to identify correctly in the field were species of *Arthroleptis*, *Hyperolius*, *Phrynobatrachus* and *Ptychadena*. The actual number of species in each of these genera was overestimated based on morphological assessment in the field, particularly during the first sampling season (Table 3). For instance, in the first field sampling period (dry season), six individuals of *Phrynobatrachus auritus* (barcode ID) were initially identified as four different species of *Arthroleptis*, and 10 individuals of *Phrynobatrachus* sp. A as six different *Arthroleptis* species. However, all individuals identified as *P*. *auritus* in the field in the second sampling period (wet season) were confirmed by DNA barcodes, except two, which were identified as *P*. sp. A based on DNA barcodes.

For most other frogs at the LMR study site (*Amietophrynus*, *Hemisus*, *Hoplobatrachus*, *Amnirana*, *Nectophryne* and *Xenopus*), the number of species identified in the field matched the number of species identifications based on barcode data, with one exception. Two species of *Nectophryne* were identified in the field, but DNA barcode analyses revealed only one (*N*. *afra*). However, the one specimen thought to be a different species (*N*. *batesi*) based on color and pattern was placed in its own COI BIN, although the ABDG grouping and 16S data placed it among other *N*. *afra* specimens, including ours and additional material from GenBank.

### Species richness estimates

**Fig 4:**
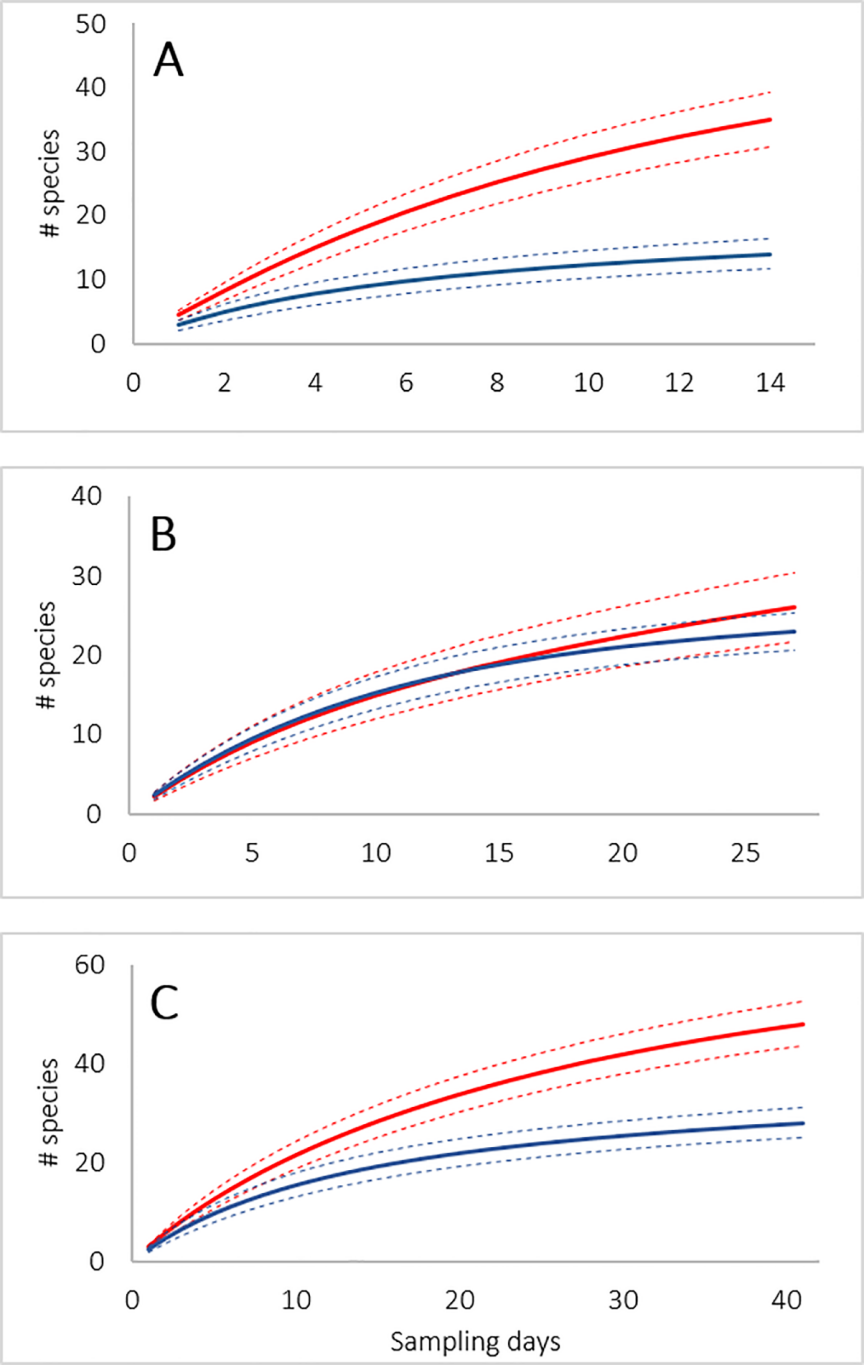
Species accumulation curves for the LMR study area in Gabon. Red lines represent species identification data from morphological assessment in the field and blue lines represent species identification data from molecular barcode operation taxonomic units for (A) the first sampling period during the dry season, (B) the second sampling period during the wet season, and (C) the combined results of both sampling periods. Dotted lines represent 84% confidence intervals.

**Fig 5:**
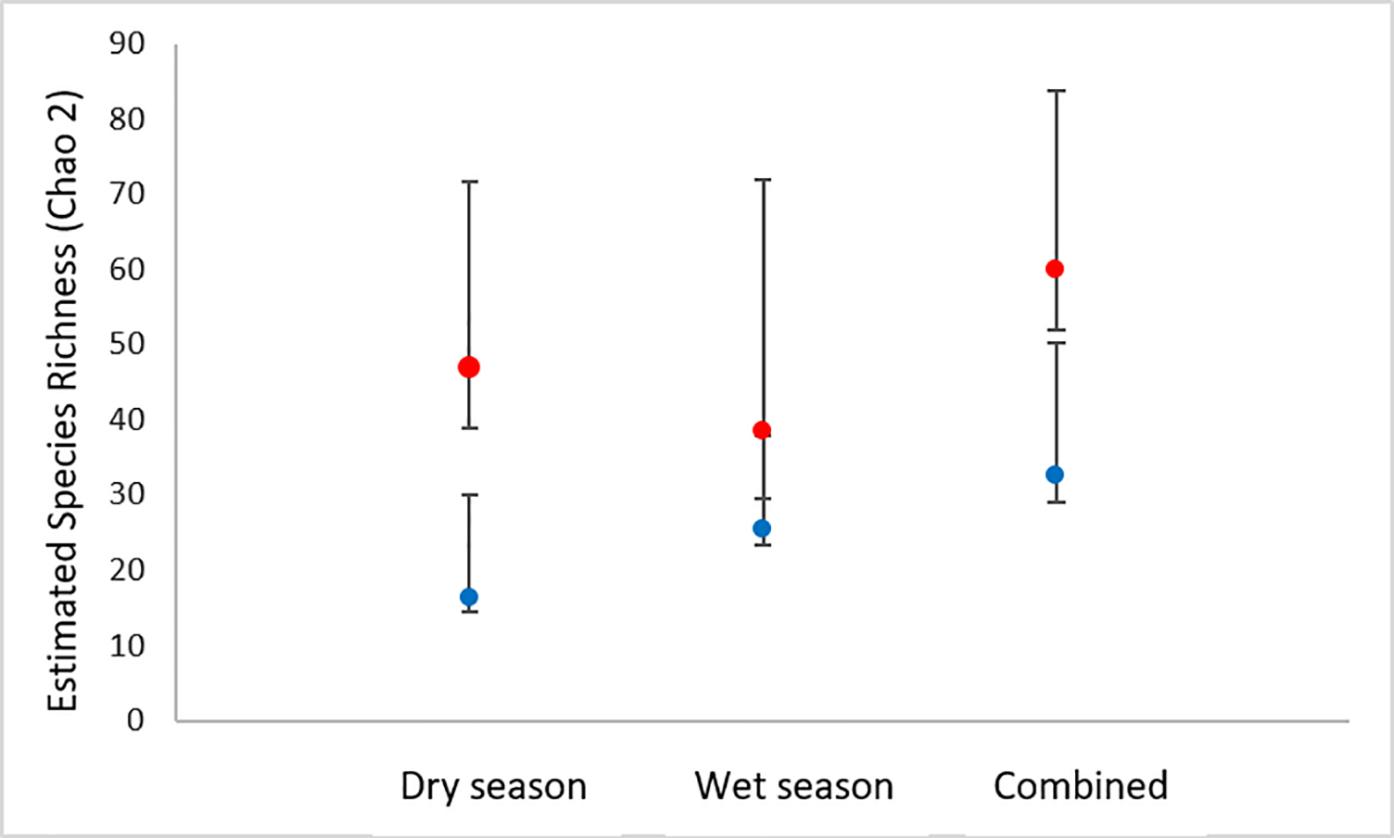
Chao2 species richness estimates for each of two sampling periods at the LMR study site and the combined results using data from morphological assessments in the field (red) and from barcode analysis (blue). Error bars are 84% confidence intervals.

Sample-based rarefaction curves show that species accumulation based on field identifications resulted in a significantly higher richness estimate in the first sampling period (dry season) than did species richness estimates generated from the results of the DNA barcoding analysis (Fig 4A). On the other hand, there is overlap of 84% confidence intervals for rarefaction estimates derived from field-based and barcode-based identifications during the second sampling period (wet season), indicating no significant difference between the two identification methods [58, 59] (Fig 4B). However, when the results from both seasons are combined, the estimated species richness for the study area based on morphological assessment in the field is significantly higher than that based on barcode data (Fig 4C). In addition, dry season and combined Chao2 richness estimates from field identifications are significantly higher [58, 59] than estimates generated using barcoding data (Fig 5).

## Discussion

A principle goal of this study was to use DNA barcoding to verify specimen identifications conducted in the field as part of a biodiversity inventory in order to provide an accurate estimate of amphibian species richness at our site. The results demonstrate that if amphibian species richness for the LMR study site had been calculated using only data resulting from morphological assessments by the field team, as is frequently the case in Environmental Impact Assessments (EIAs) in developing economy countries, species richness would have been overestimated by approximately 70%. This level of error would likely have had significant reprecussions for conservation management decisions based on the data. DNA barcoding allowed us to quickly identify the errors and adjust the species richness variable in the inventory dataset.

The second objective was to increase the publicly available DNA barcode data for west Central African amphibians. Given the paucity of preexisting data (particularly for COI), this study makes a substantial contribution toward building a comprehensive barcode library for west Central African amphibians that will be useful to current and future research in conservation, ecology, and evolution [87]. These data provide a solid foundation by which additional taxonomic identifications of African amphibians can be made, and to which sequences from many as of yet unsampled species can be added.

Taxonomic resolution to the level of species is necessary to accurately apply performance indicators and biodiversity metrics in ecosystem assessment and monitoring. DNA barcoding not only revealed overestimated field-identified amphibian richness at the LMR site, but also allowed us to more accurately assign taxonomic identities than was possible with morphology alone by comparing our sequences to those in GenBank. The ability to assign accurate identities allows greater precision in evaluating amphibian diversity, and a DNA barcode library provides a reference against which future field identifications can be confirmed. Combined, these advances will allow us to more adequately track changes in populations over time and to set goals for maintaining and restoring populations in recently disturbed areas. Specimen identification, through DNA barcoding, can provide the resolution necessary to be able to identify community-wide changes immediately and to respond accordingly within an adaptive management framework.

Given uncertain taxonomy, as well as taxonomic advances that post-date the field sampling, it is not surprising that some species identifications based on morphology were not corroborated with barcoding results. However, misidentification of some specimens at the generic level and the overall discord in the total number of species detected is more surprising. This discrepancy in species richness estimates using morphological data and barcode data was much reduced in the second sampling period when a more experienced amphibian biologist participated in the field sampling. Given the rate at which habitat is being altered and the need for biodiversity assessments in threatened areas, it will not always be possible for a highly trained researcher to accompany biological inventory teams. Unfortunately, it has been our experience that biodiversity assessments conducted, for example, as part of EIAs in some parts of the world frequently utilize younger, less experienced field teams to limit costs, or because more experienced teams are not available. The present study suggests that such decisions could have negative implications for the accuracy of results of these surveys. In such instances, we recommend that integrated surveys always be used, taking care to collect lasting, detailed records through specimens and tissue samples and, where possible, acoustic recordings so that DNA barcoding and other integrative tools can be used to confirm identifications.

While cryptic diversity, like that found among *Arthroleptis* [68] and *Ptychadena* [84], affords a well-recognized foundation of confusion for field taxonomists [20], it is phenotypic variation within *Phrynobatrachus* [21] and *Hyperolius* at the LMR study area that posed the most significant challenges for our parataxonomists and experts, alike. DNA barcoding allowed us to point out those genera that caused the most identification challenges in the field. Our findings should be used to supplement others working on the taxonomy of these groups by providing additional geographic and taxonomic sampling of these problematic taxa. Voucher specimen collections are encouraged for future proper taxonomic treatments. The ultimate goal would be to produce specialized field guides that can help to distinguish morphological characters of species within those genera proven to cause the most confusion.

We cannot overemphasize the value parataxonomists provide in support of research and conservation [88]. One of the challenges to their capacity for research stems from the extent of scientific training and tools available to support their efforts. Refined species identification based on DNA barcoding can act as an important adaptive tool to support parataxonomists in long-term studies. Through barcode-confirmed and revised identifications, and examination of previously collected specimens, parataxonomists can learn to recognize the morphological characters that distinguish species at their site, rather than basing their identifications on field guides developed using specimens from other geographic regions, which may vary from those observed in their local study areas. Thus, verification through DNA barcoding provides an important opportunity to reduce the potential for error in data collection and to train local scientists, allowing them not only to improve their own taxonomic skills, but ultimately raising the taxonomic quality of biodiversity inventories over time.

Although we were not able to resolve all taxonomic issues with the current barcode data, we were able to discern clades, which may represent taxonomic units that likely will require future work to discern their identity or to describe as new species. For example, *Hemisus* specimens from Gabon and RC, initially identified as *H*. *perreti*, formed distinct clades, recognized here as two species. In addition, seven species of *Arthroleptis* were identified, but we were only able to assign species names to five of them, leaving two temporarily identified as *Arthroleptis* sp. A and B.

While DNA barcoding is certainly useful for discerning the number of OTUs at a site, it is important to be able to assign accurate scientific names to those OTUs for barcoding to be most useful in conservation biology. Databases such as GenBank and BOLD can help with assigning scientific names to taxonomic units, but sequences in these databases must be correctly identified to do so. Unfortunately, genetic databases such as GenBank and others are often wrought with taxonomic misidentifications [89–92]. For example, many records in GenBank are misidentified, particularly for anurans, either through original designations, or because our current understanding of the taxonomy has changed. In addition, currently only the original researchers who submit sequence data to GenBank are allowed to update taxonomic identification, even if other researchers (or holding institutions) have subsequently re-identified the specimen. Additionally, many sequence records lack museum catalogue numbers and locality information (currently not a requirement, but a mere option in GenBank), having only minimal data, such as a personal field number. Poorly curated records make it increasingly difficult for researchers to be able to compare their material with reference material in GenBank. While voucher and locality information can sometimes be found in publications or appendices therein, this is not always the case. If specimens and localities cannot be tracked back from sequences, they are essentially useless; in fact, they create a sort of DNA barcode pollution—sequences with questionable or incorrect identifications that cannot be updated as our taxonomic and geographic knowledge of biodiversity continues to grow. Conclusions reached in studies supported by sequences from publicly available DNA databases will only be as accurate as the identifications provided in the database. Here, we make a plea to researchers to actively update the taxonomy and metadata of specimens for which they are responsible and thereby maintain a useful database of genomic information. We also suggest that GenBank allow third-party annotations to incorporate novel information published by those that did not originally submit the sequence data.

Though many sequences exist in GenBank for comparative purposes at the generic-level, taxonomically relevant sampling at the species-level was sparse for groups in this study, with geographic coverage being even more limited. For this reason, we used simple analytic approaches, such as the alignment method in Geneious in which we did not remove ambiguous (loop region) characters, and used fast (neighbor-joining) tree building algorithms. While more sophisticated species delimitation methods are available, such as SpeciesIdentifier [93] and the Generalized Mixed Yule Coalescent model (GMYC) (e.g. [94]), these methods are more effective at identifying species limits when comprehensive taxonomic treatments are being conducted with sufficient geographic and taxonomic sampling in the group of interest [94], preferably with the inclusion of nuclear markers [85]. On the other hand, the COI BIN [50] or ABGD [52] methods and neighbor-joining trees generating clades [95] can provide quick assessments of haplotype diversity when taxonomic and geographic samples are limited. These results must be carefully interpreted, and initial identifications (e.g. “sp. A”) can be useful for distinguishing unknown lineages [96]. In the current study, the BIN and ABGD methods largely agreed, and only differed when what we considered a single species was placed into multiple BINs, but often only in one ABGD group (Table 2). Further geographic sampling is needed in these groups and the COI barcoding methods are impeded by hybridization and incomplete taxonomic and geographic sampling [35]; hence, our cursory methods and more conservative decisions to maintain more widely distributed, genetically variable species in several of these cases. This method of identifying species, even if un-named (e.g. sp. A), moves taxonomic knowledge forward. As new material is added to BOLD—as the ‘BINs of Life’ are filled—we will eventually capture the sequence variation of all known species. As more individuals per species are added, more sophisticated species delimitation methods can be conducted. The BOLD system allows users to generate NJ trees and haplotype networks for specimens they chose to include, for more taxonomic and geographic coverage, as the database becomes more populated. We propose this method to conservation biologists, ecologists and parataxonomists working on problematic organismal groups with limited geographic sampling, sparse molecular representatives, and comparative morphological material available, but who may also have access to DNA barcoding resources.

It is important to note that barcoding presents various pitfalls, and precautions need to be taken when applying the methodology to ecological and conservation biology studies (e.g. [39, 97–100]). One logistical complication involving barcoding is the cost [97]. In the current study, we worked with private industry and argue that the costs associated with barcoding are worth the investment by companies required to do biodiversity assessments, as it will enable them to provide accurate information to local and national governments regarding potential impacts of infrastructure projects. For example, if 60 amphibian morphospecies were inventoried during an EIA, and 10 specimens of each were barcoded [101] at $5/specimen [97], the cost to the company would be approximately $3,000. In the event that species in some genera prove particularly difficult to identify (as was the case in the current study), it may be necessary to barcode additional specimens from those groups. The addition of 200 specimens would add about $1,000 to the overall budget. Although difficult to estimate, the average cost of a typical EIA appears to be between $250,000 and $2 million USD, depending on the size and scope of the development project [102]. Relative to the overall cost, integrating a barcoding component to an EIA could be a minor (1% or less of total cost), yet valuable investment for most projects. Additional taxonomic groups (e.g. insects, plants, birds) could also be considered for relatively little added cost.

Some may argue that DNA barcoding is an unrealistic method for specimen identification in developing countries. One claim is that a lack of access to molecular tools and equipment will prevent DNA barcoding from becoming routine. However, technological advances in molecular equipment now make it possible to extract DNA, conduct PCR and electrophoresis, and even sequence DNA in even the most remote locations, and the costs are decreasing [103]. We encourage colleagues to continue efforts already underway and to initiate new endeavors to build capacity in molecular analysis in countries with developing economies to ensure that these methods are accessible in any region. Another argument is that new international legislation has made conducting cross-border genetic research impossible. While international legislation such as the Nagoya Protocol may change “business as usual”, it does not preclude research using genetic material, rather it ensures that users obtain prior informed consent before accessing genetic resources (in this case, the collection of specimens) and establish mutually-agreed terms, including sharing of benefits (monetary or non-monetary), between the user and providing country. Arguably, it has improved capacity for genetic research in some countries because of increased collaboration between nations within the providing countries borders. While it may take some countries time to integrate fair and equitable sharing of benefits as a result of the access of use of genetic resources, we do not agree that it will prevent DNA barcoding from becoming a useful tool for biodiversity in developing countries, and in fact suggest that it provides a route for increased in-country capacity building, and between-country data exchange and collaboration.

In this study, we experienced the unnecessary and unproductive dichotomy between the fields of molecular taxonomy and biodiversity inventory [2]. Molecular taxonomic studies and biodiversity inventories have different agendas designed to answer distinct questions; however, we suggest that the two are actually complementary and there is much room for productive collaboration. While molecular taxonomy sets out to systematically sample entire geographic distributions of species complexes (the best studies require meticulous planning, review of existing collections, and selection of geographic and taxonomic gaps in need of sampling), biodiversity inventories are frequently carried out under “emergency” situations to provide data for evaluating biodiversity in the context of unforeseen and/or imminent local threats. Nevertheless, given that biodiversity inventories often sample poorly known areas with limited access, taxonomists should consider these as opportunities to acquire additional information necessary to resolve complex systematic questions, through added specimens, sequence data, and potential sources of DNA for further analyses. After incorporating this information into their work, molecular taxonomists can, in a timely manner, update guides and databases with improved taxonomic knowledge, taking care to identify distinguishing morphological characters that will aid inventory specialists in their work. Researchers and parataxonomists conducting biodiversity inventories, in turn, should recognize their responsibility in collecting data useful to molecular taxonomists alongside their own. This includes careful preparation and storage of specimens and tissues, detailed and precise recording of metadata associated with these vouchers (e.g. color notes, reproductive behavior, etc.), reference photographs and acoustic recordings, and making these data available to taxonomists by depositing the specimens in well-curated and open collections. If conscientiously implemented by both fields, this process will create a productive feedback loop to improve knowledge of species as well as their conservation and management.

While many researchers continue to work tirelessly to untangle African amphibian taxonomy, the current rate of economic development continues unabated, and forces decision makers to design strategies for biodiversity conservation often based on limited or no data. While traditional surveys can certainly be useful for initial estimates of species richness at a given site, and these estimates can guide conservation strategy, it is clear that the potential to over- or underestimate richness is real, and may be heavily impacted by the experiences and training of the inventory team, as well as the quality of resources available for identification. Our study shows DNA barcoding can be an incredibly useful tool to help support ecologists, conservation biologists and parataxonomists alike in the field by providing a rapid assessment of species diversity. We also argue that a reference barcode library can reduce significantly the number of identification errors in any given biodiversity data set. While DNA barcoding alone may not be able to resolve taxonomy, it does provide a framework to assess species richness more accurately through the identification of OTUs and should be integrated without hesitation into future biodiversity assessments.

Furthermore, we advocate that future comprehensive taxonomic studies incorporating molecular data be conducted on the problematic groups of west Central African anurans and offer some advice in doing so. Studies can make use of our summary of the current taxonomic understanding for each group investigated herein. The tentative identifications and unknown lineages identified in this study can be used to investigate whether the specimens we studied represent new species, or previously identified un-described species. Future taxonomic work should also strive for more comprehensive sampling, both taxonomically and geographically, and the use of more rigorous methods of analyses, such as morphological comparisons with type material, original species descriptions. Incorporating our recommendations will result in additional progress within each group and our knowledge of west Central African anuran taxonomy will continue to improve.

## Supporting information

S1 Fig Arthroleptidae 16S neighbor-joining tree. (PDF)

S2 Fig Bufonidae 16S neighbor-joining tree. (PDF)

S3 Fig Conrauidae and Hemisotidae 16S neighbor-joining tree. (PDF)

S4 Fig Dicroglossidae 16S neighbor-joining tree. (PDF)

S5 Fig Hyperoliidae 16S neighbor-joining tree. (PDF)

S6 Fig Phrynobatrachidae 16S neighbor-joining tree. (PDF)

S7 Fig Pipidae 16S neighbor-joining tree. (PDF)

S8 Fig Ptychadenidae 16S neighbor-joining tree. (PDF)

S9 Fig Pyxicephalidae, Ranidae and Rhacophoridae 16S neighbor-joining tree. (PDF)
