# Supplementary figures and images for: How many species and under what names? Using DNA barcoding and GenBank data for west Central African amphibian conservation
Source: PLoS One. 2017 Nov 13;12(11):e0187283. doi: 10.1371/journal.pone.0187283 (PMC5683629; doi:10.1371/journal.pone.0187283)

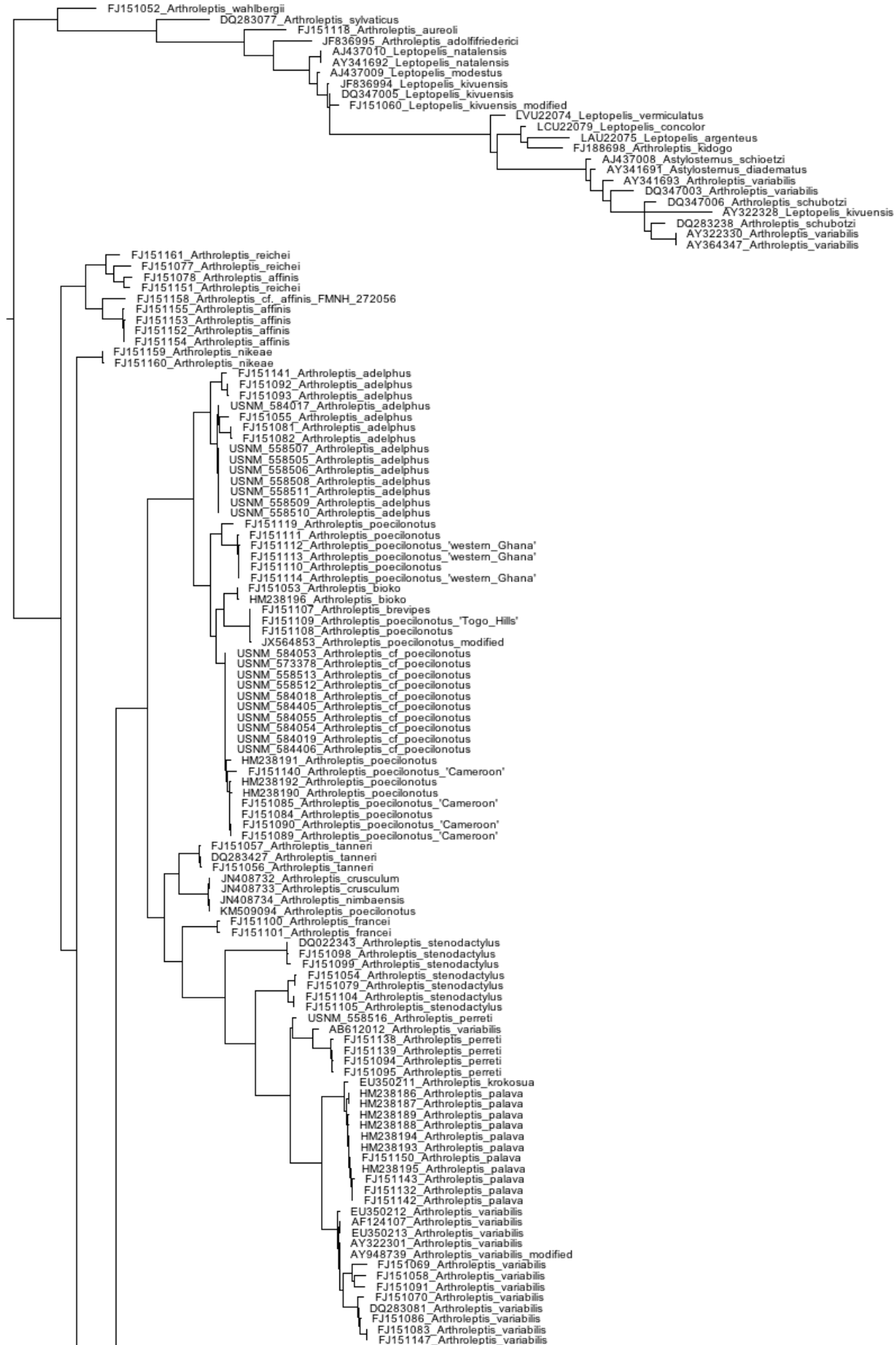

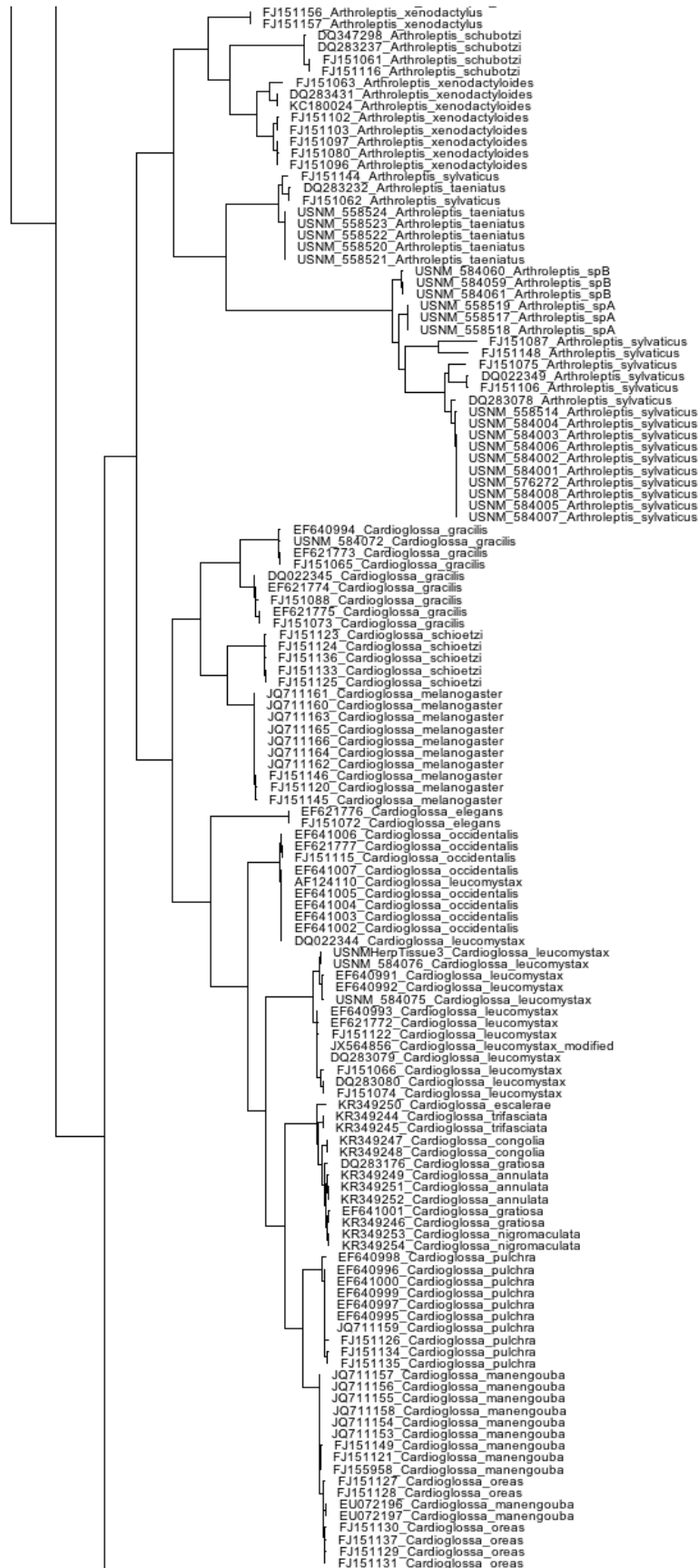

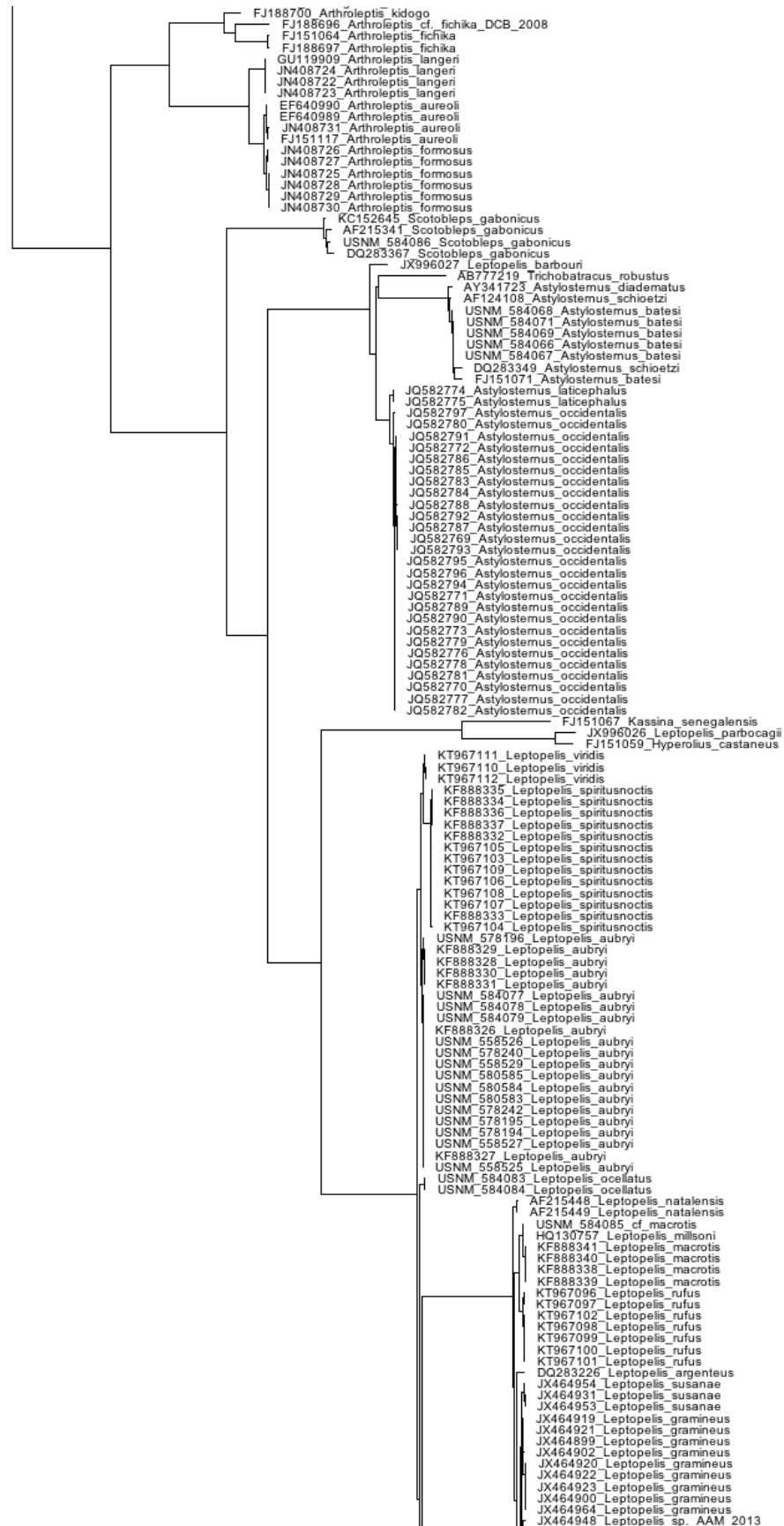

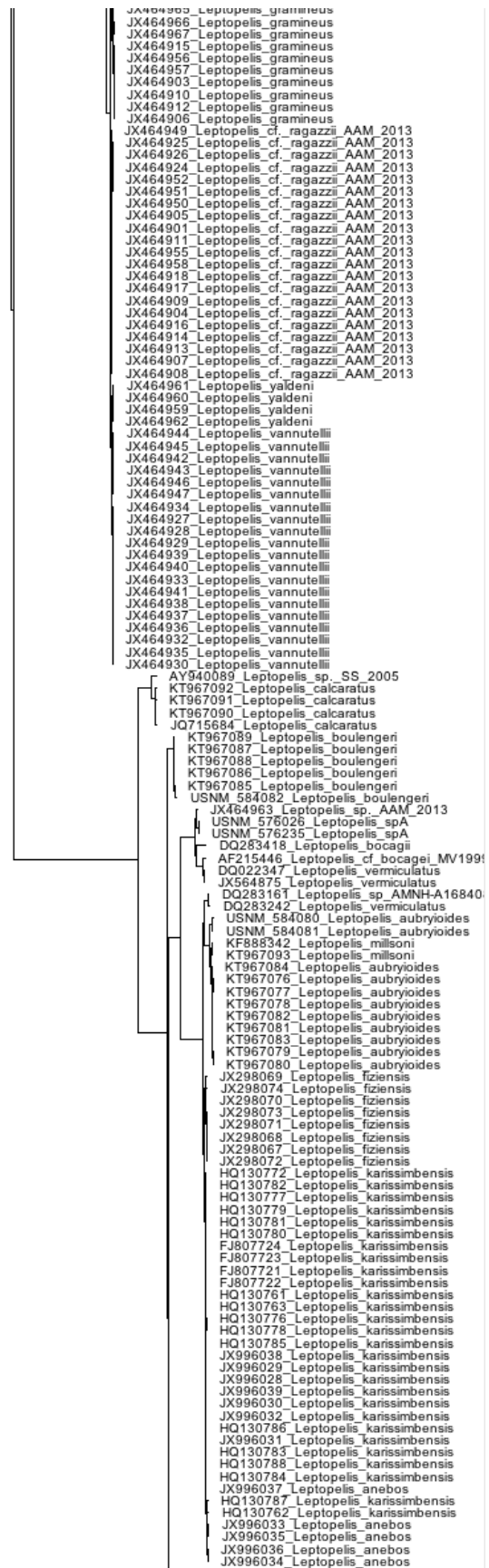

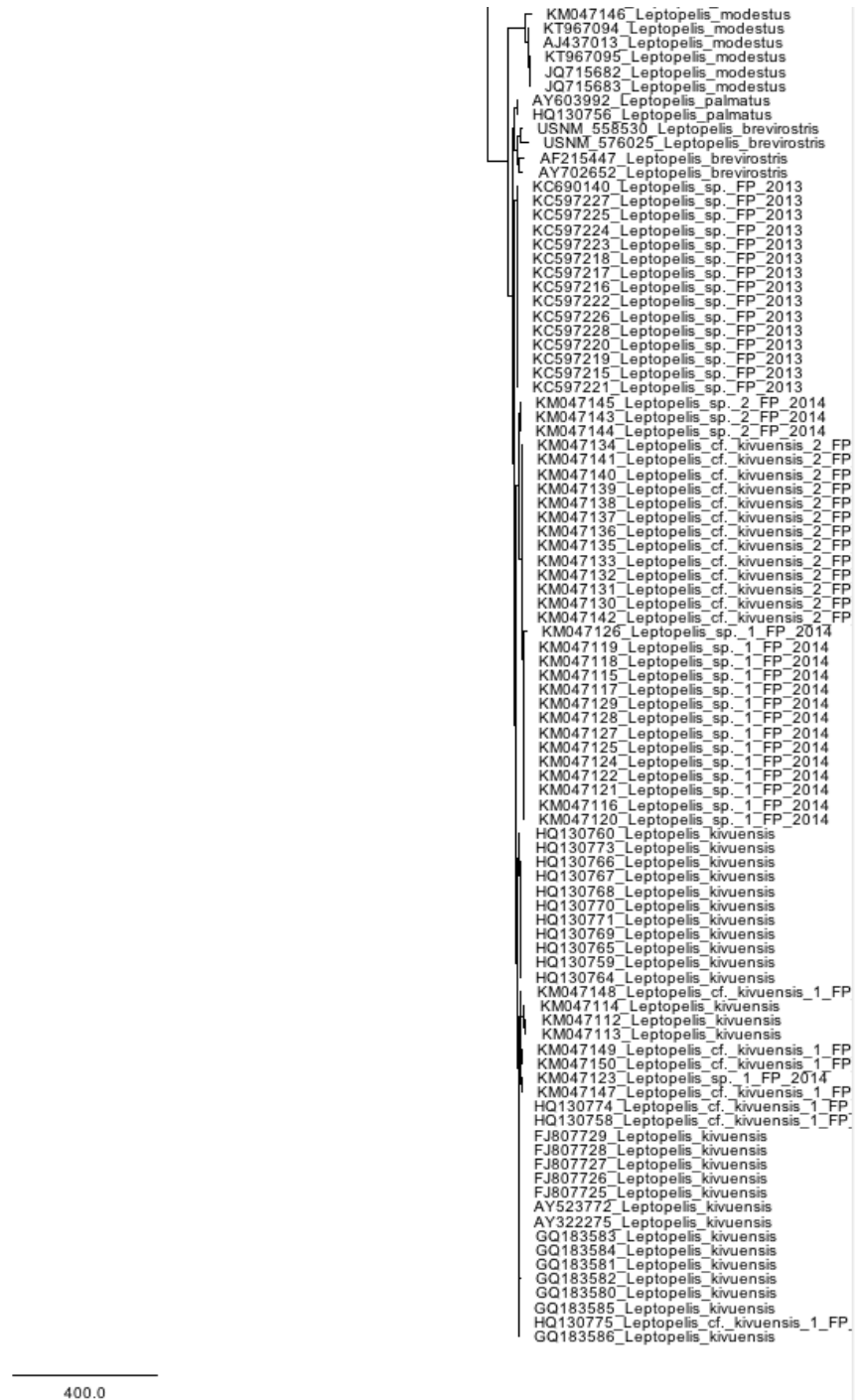

Supplement: S1 Fig — (PDF) [file pone.0187283.s001.pdf]

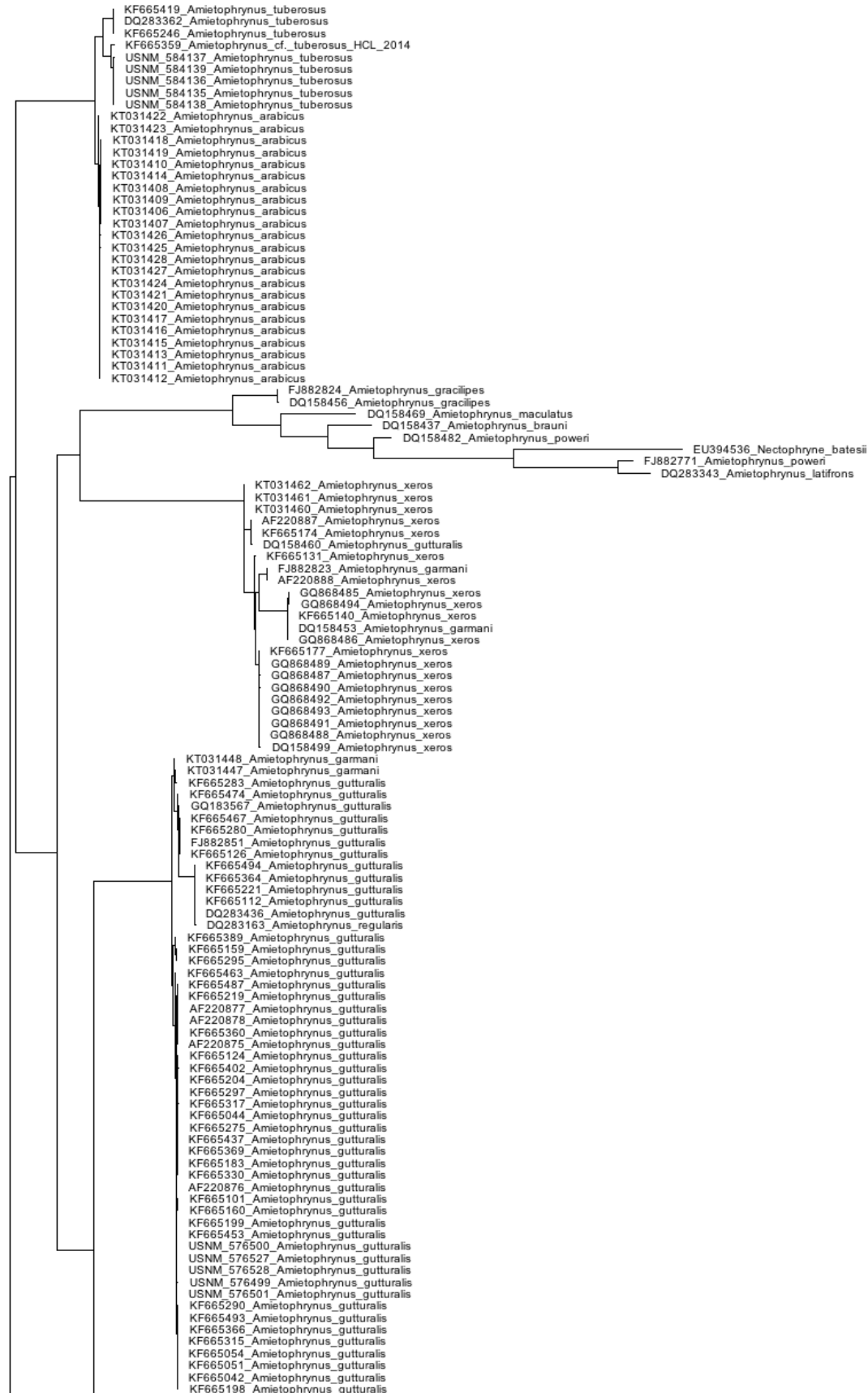

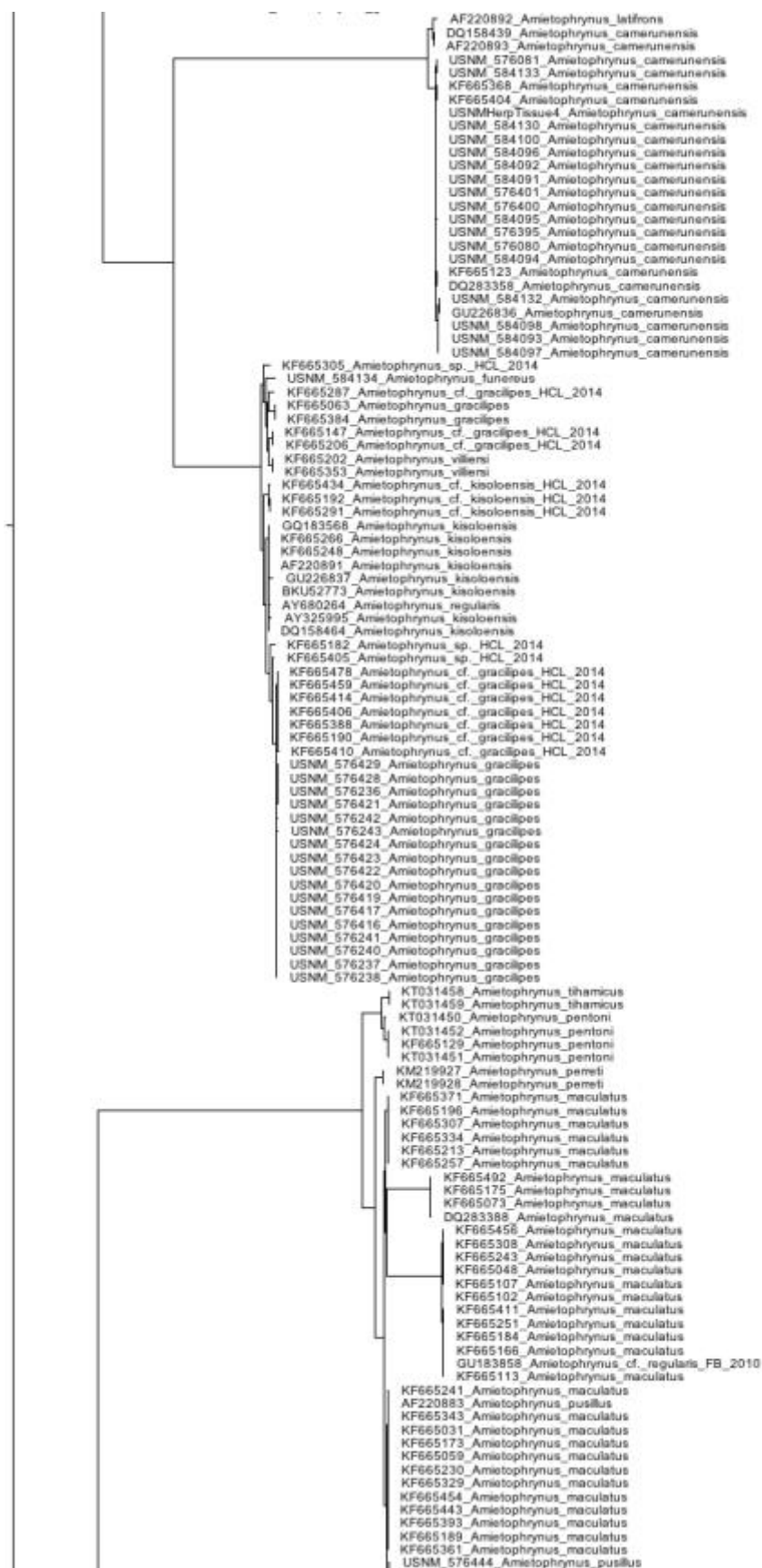

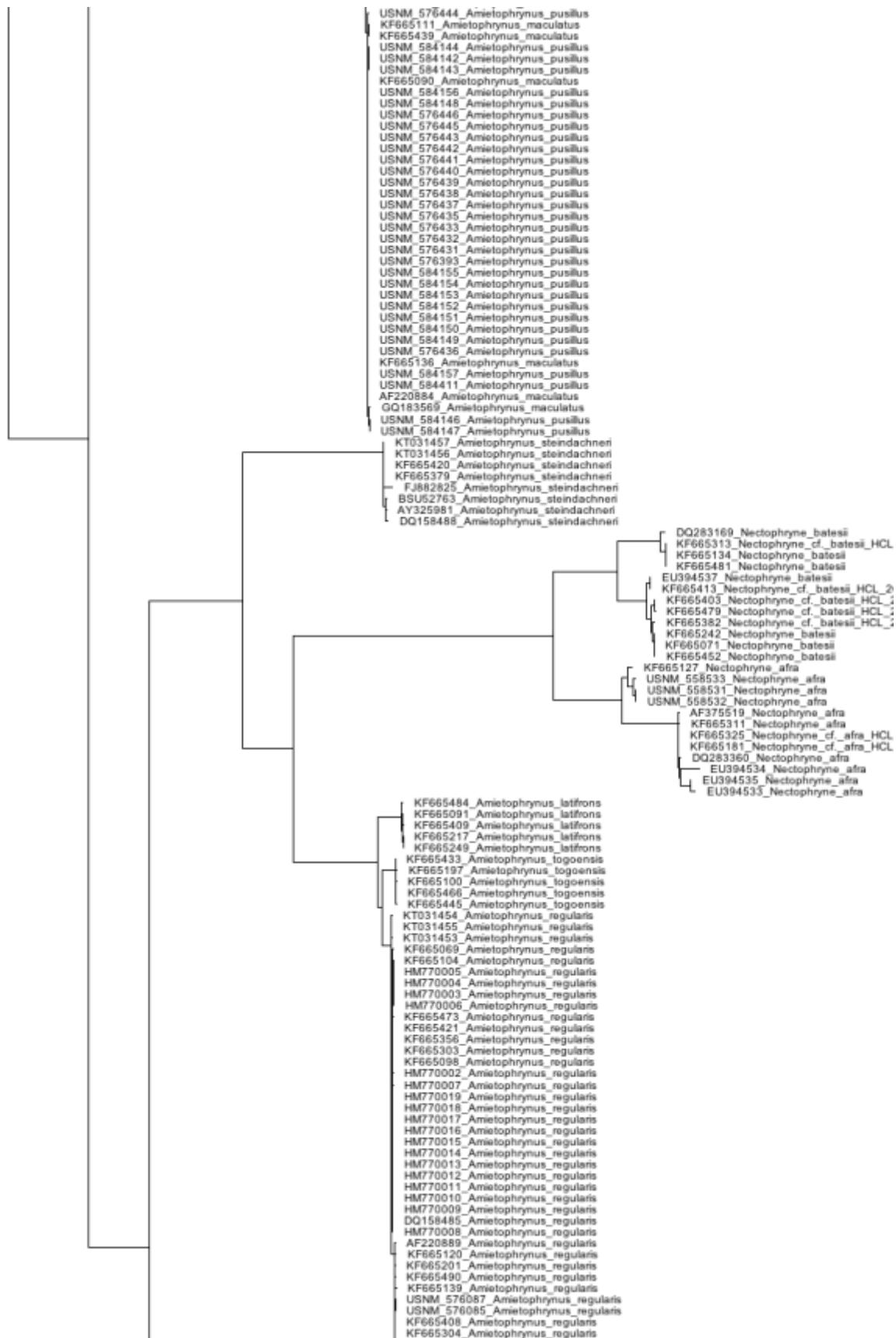

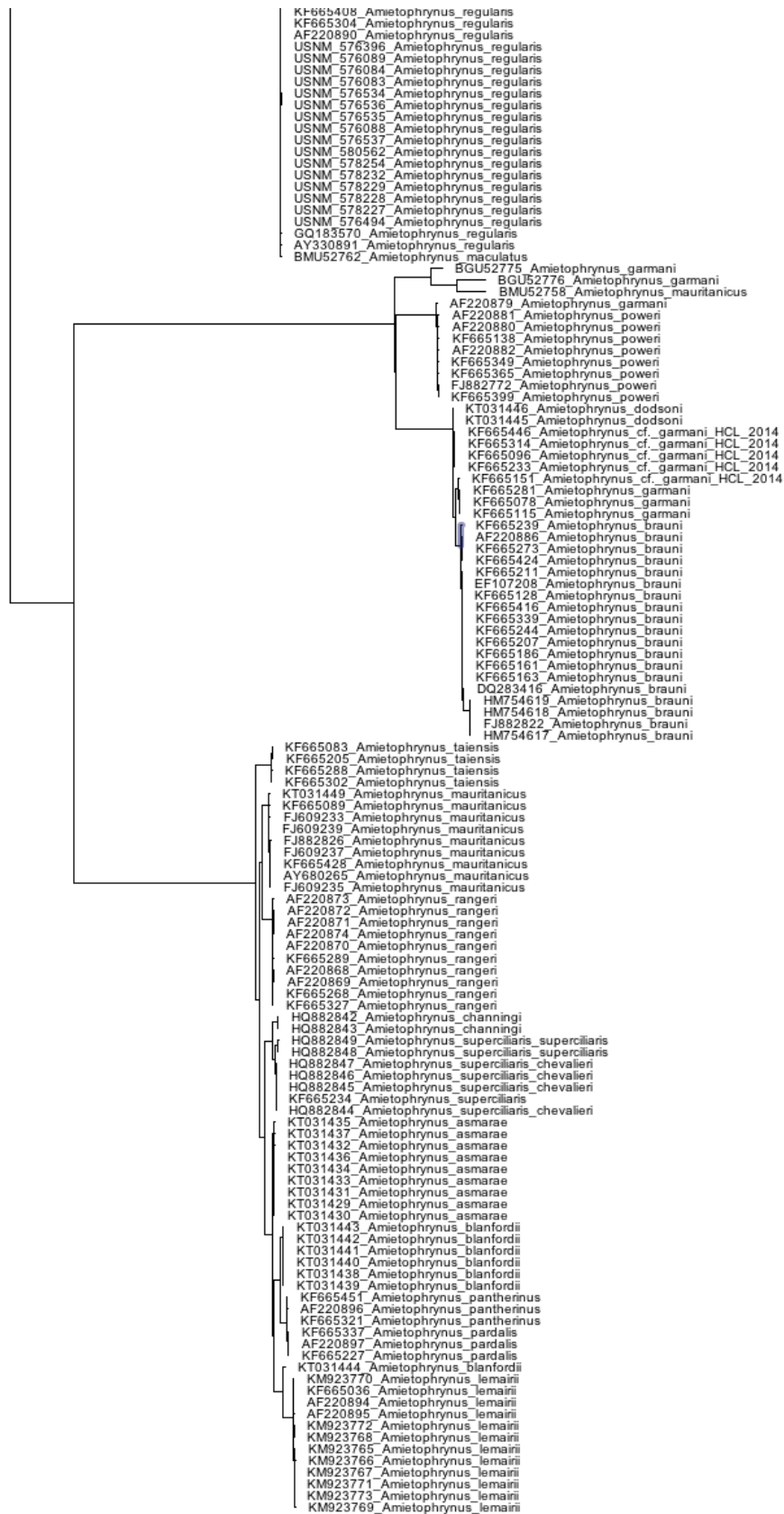

300.0

Supplement: S2 Fig — (PDF) [file pone.0187283.s002.pdf]

Deichmann et al., 2017. Supporting Information Figure 3. Conrauidae and Hemisotidae 16S.

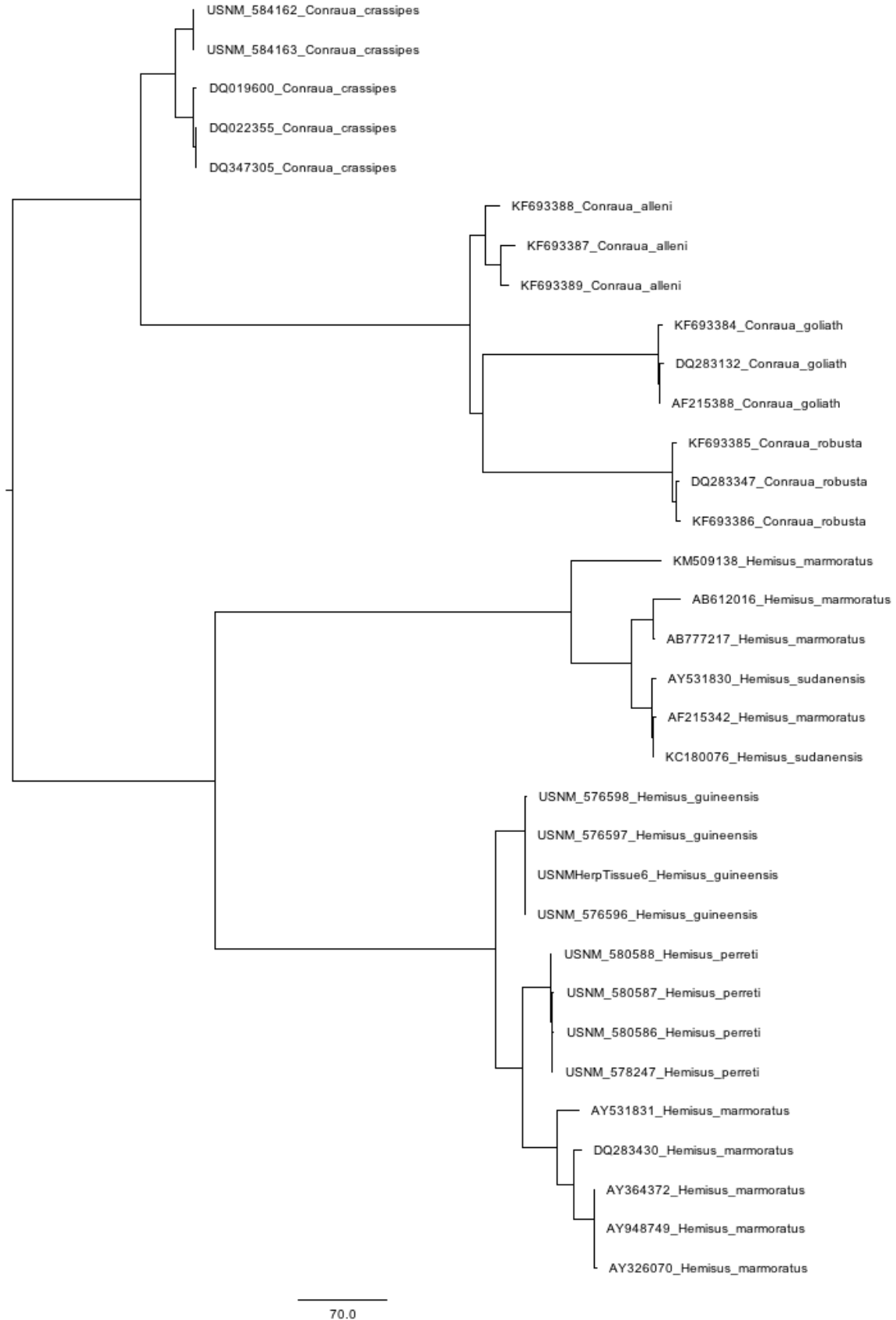

Supplement: S3 Fig — (PDF) [file pone.0187283.s003.pdf]

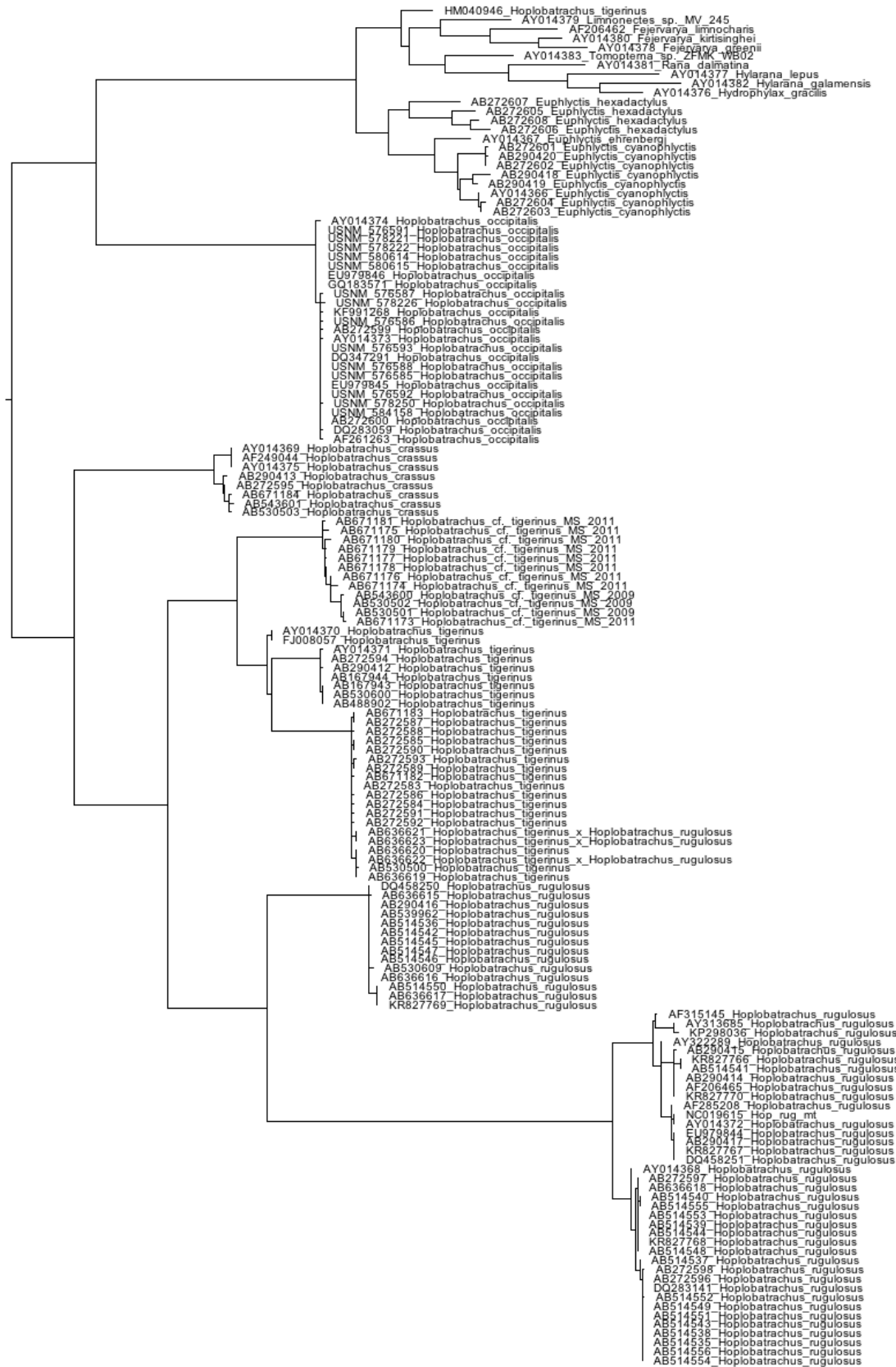

Supplement: S4 Fig — (PDF) [file pone.0187283.s004.pdf]

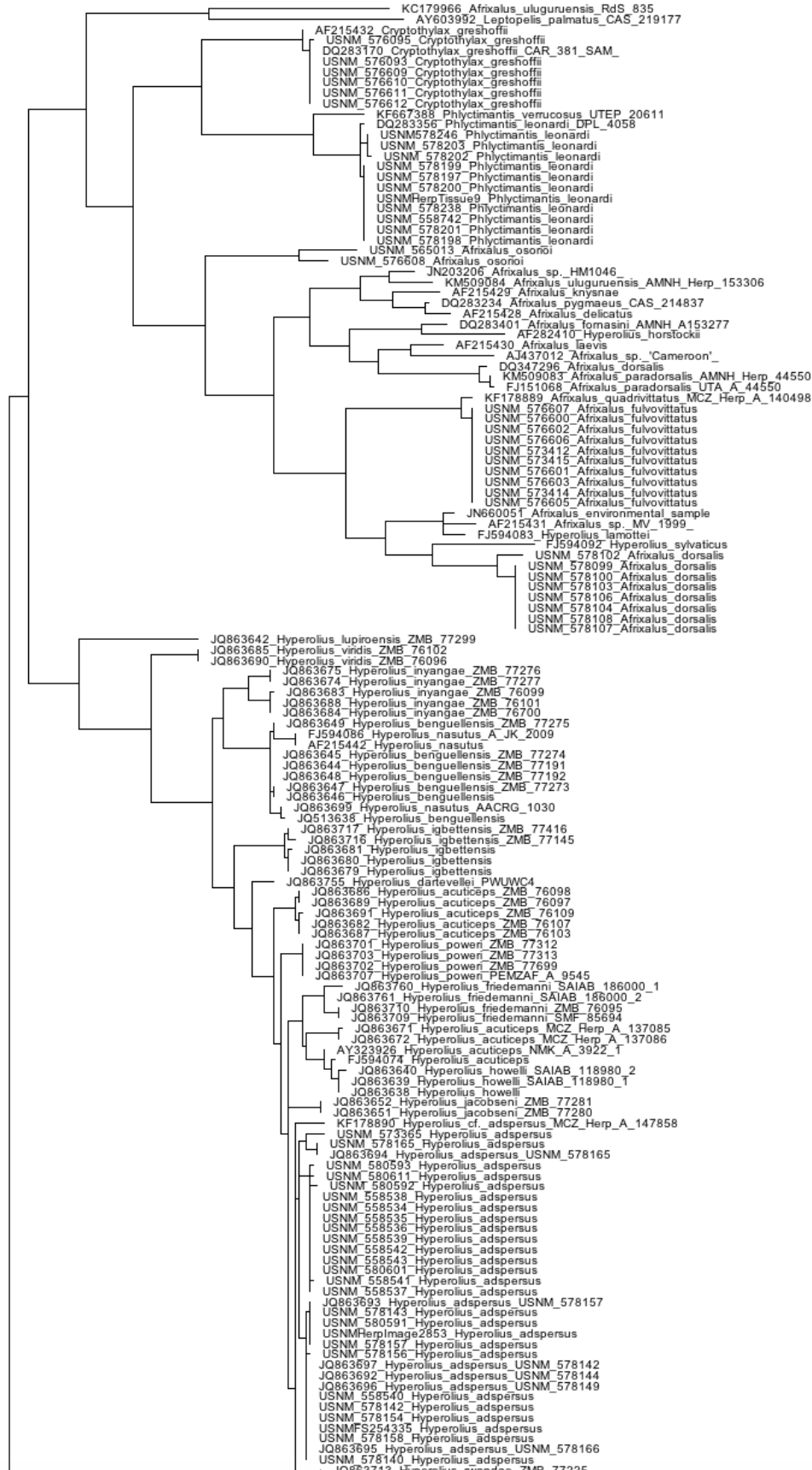

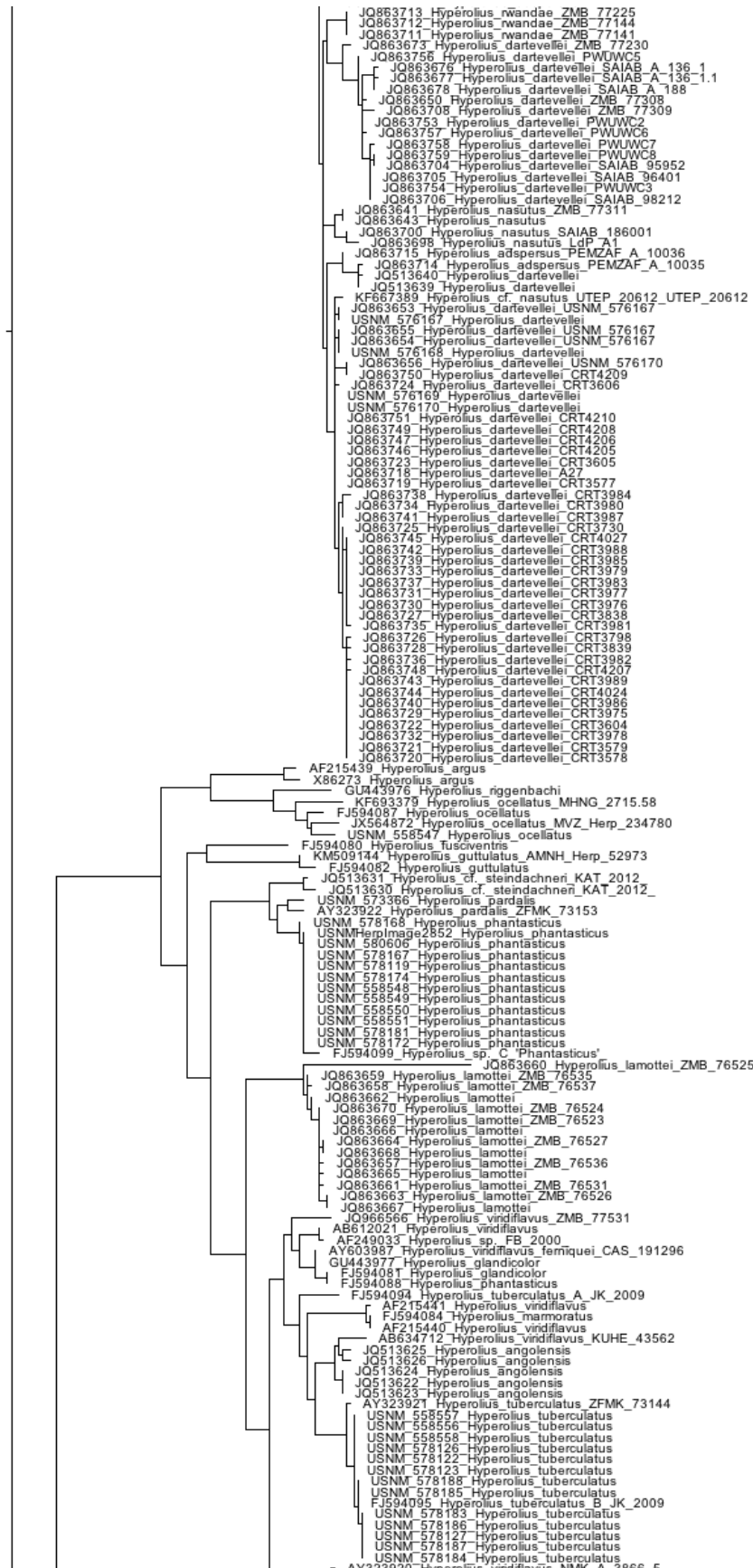



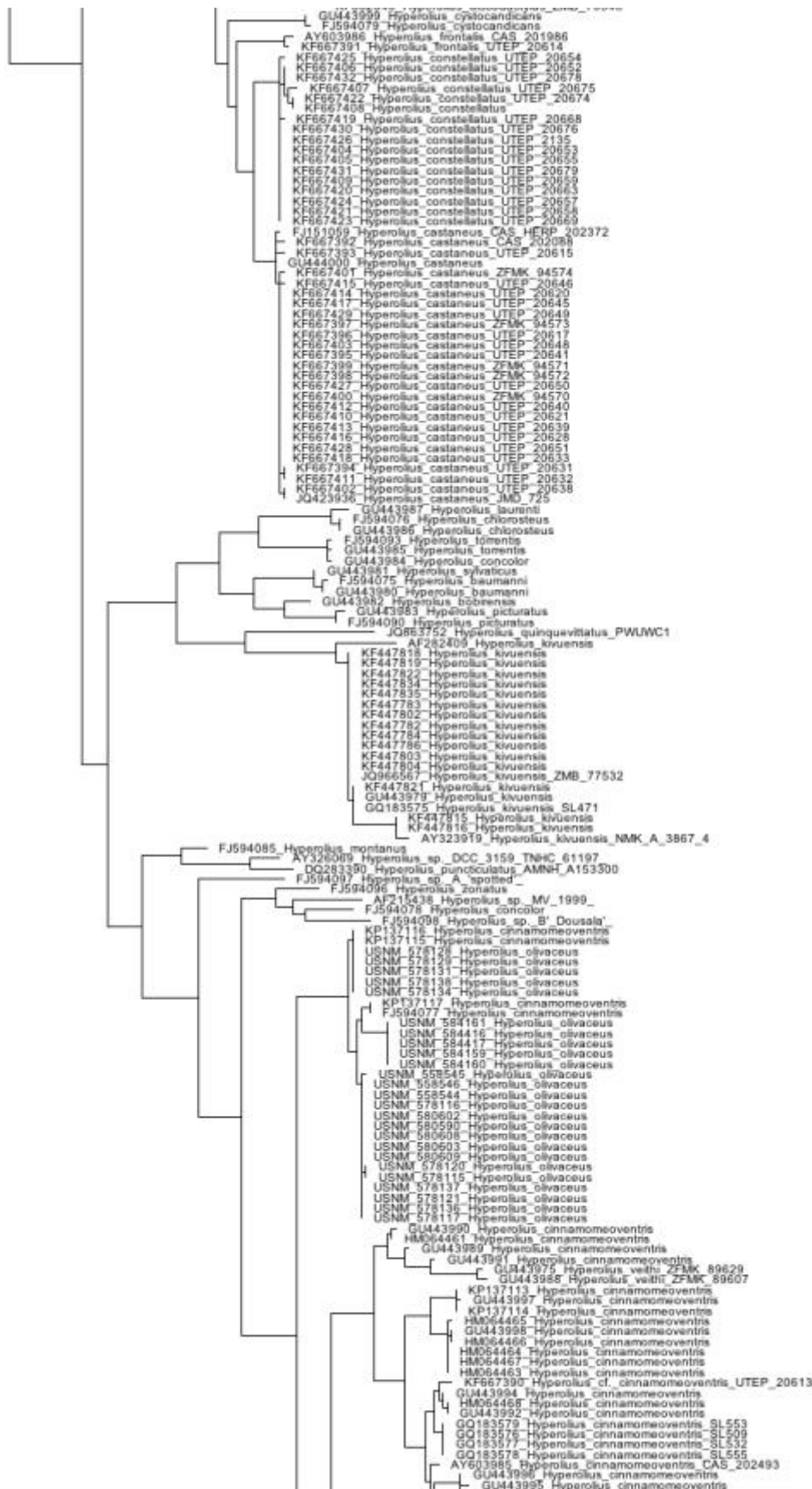

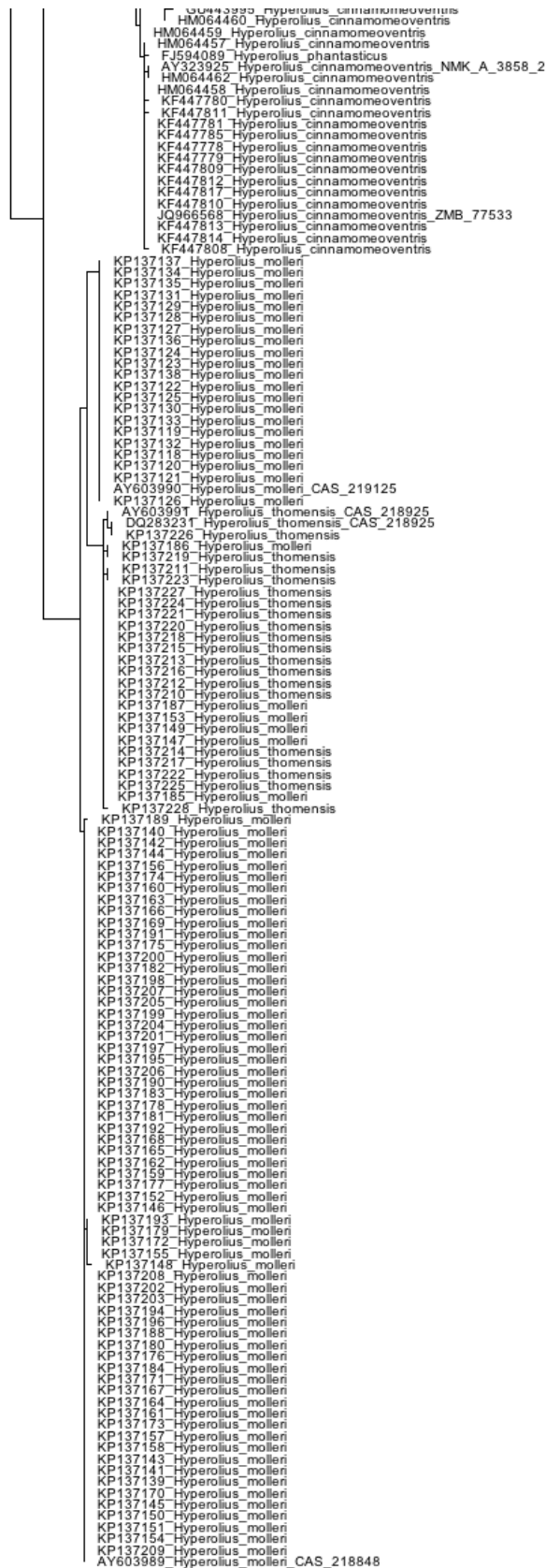

Supplement: S5 Fig — (PDF) [file pone.0187283.s005.pdf]

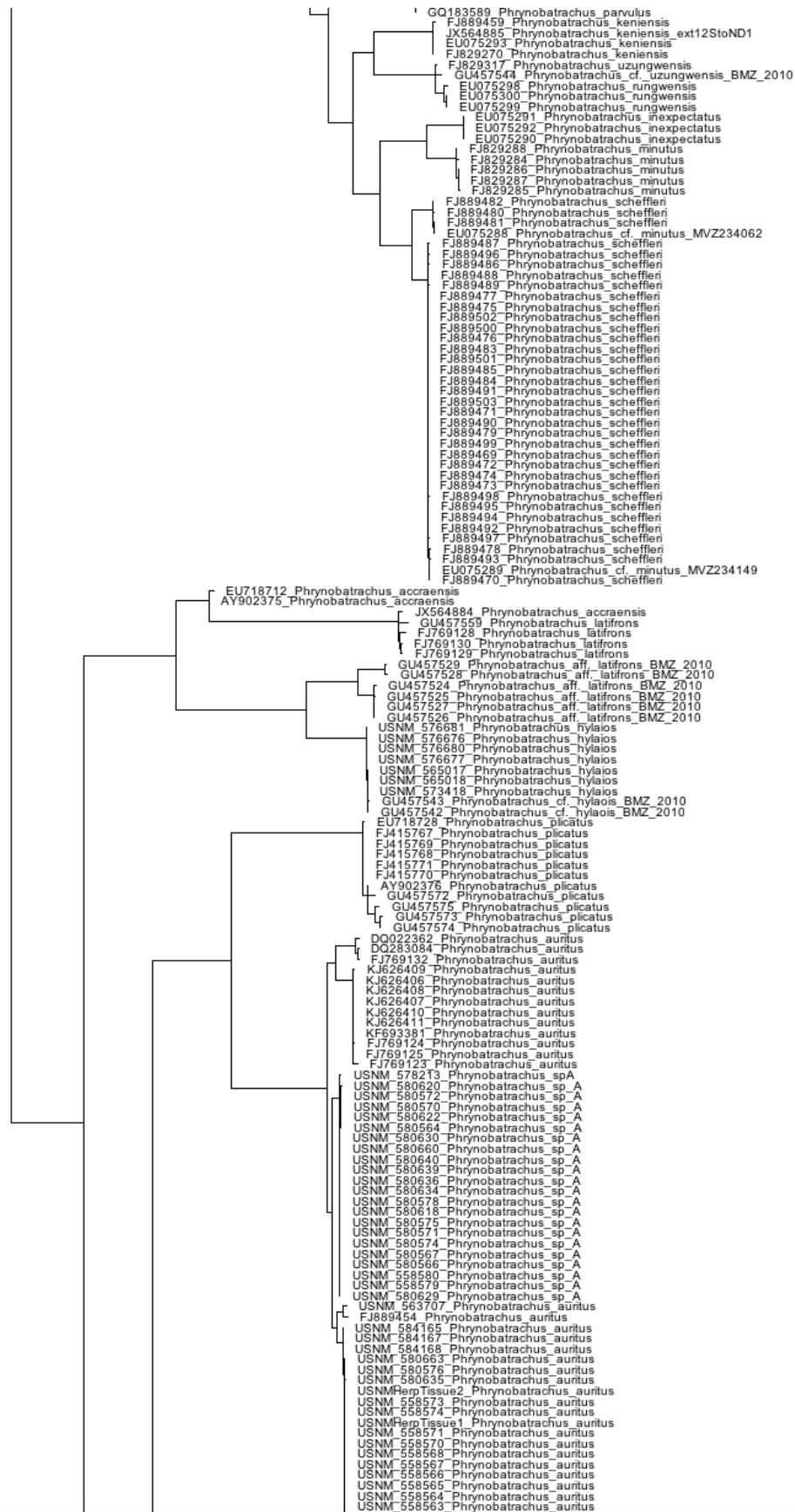

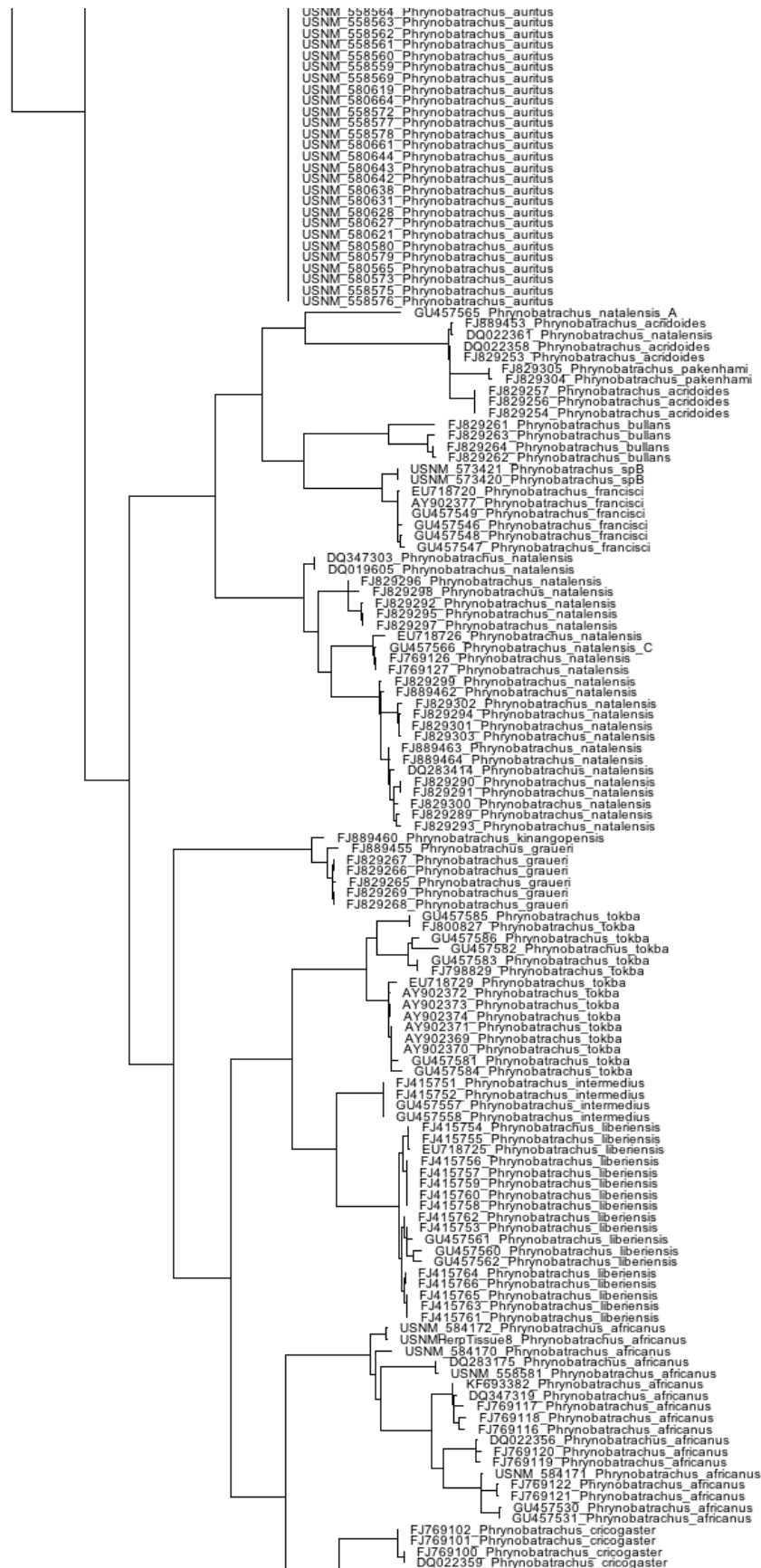

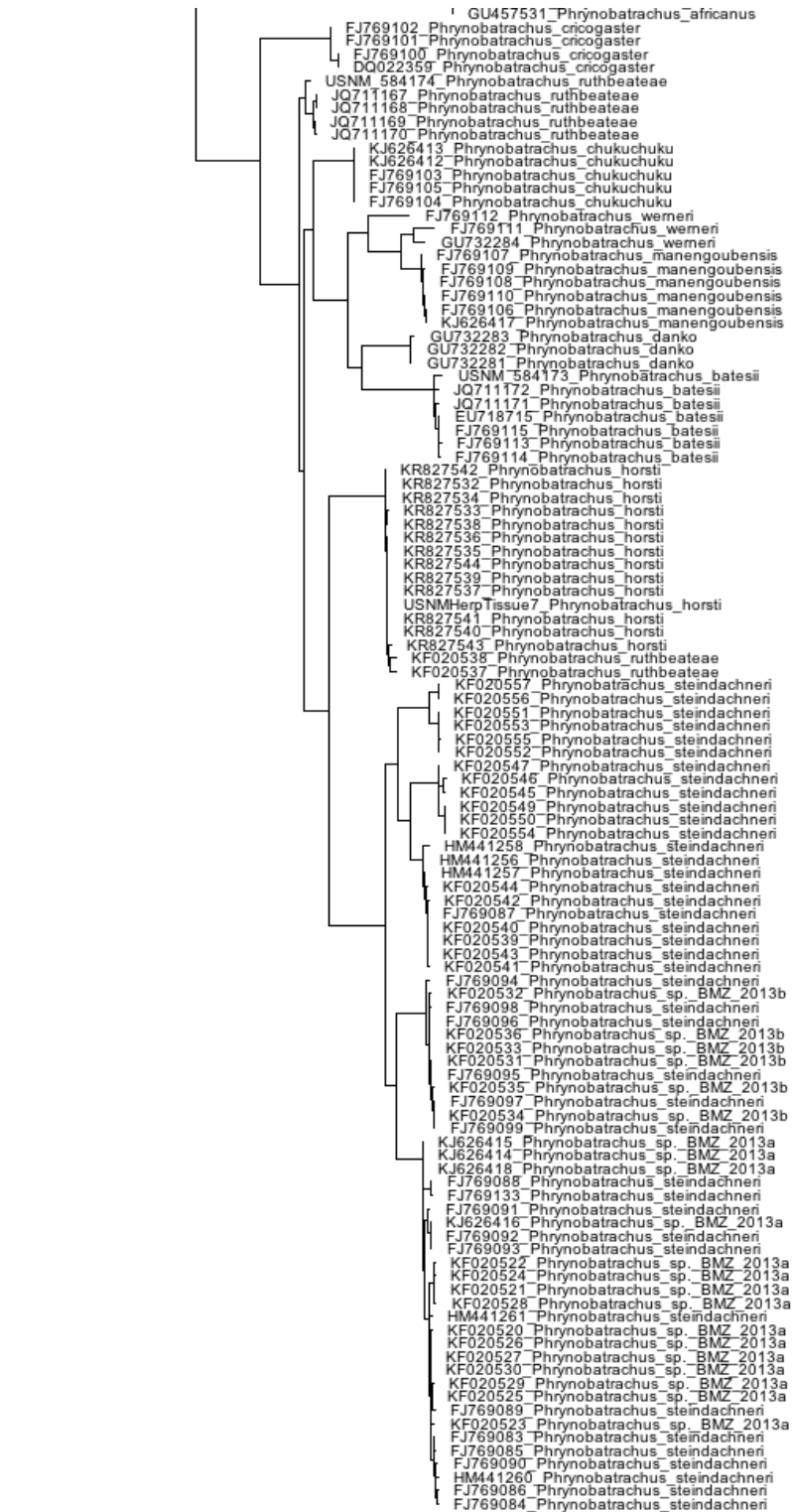

200.0

Supplement: S6 Fig — (PDF) [file pone.0187283.s006.pdf]

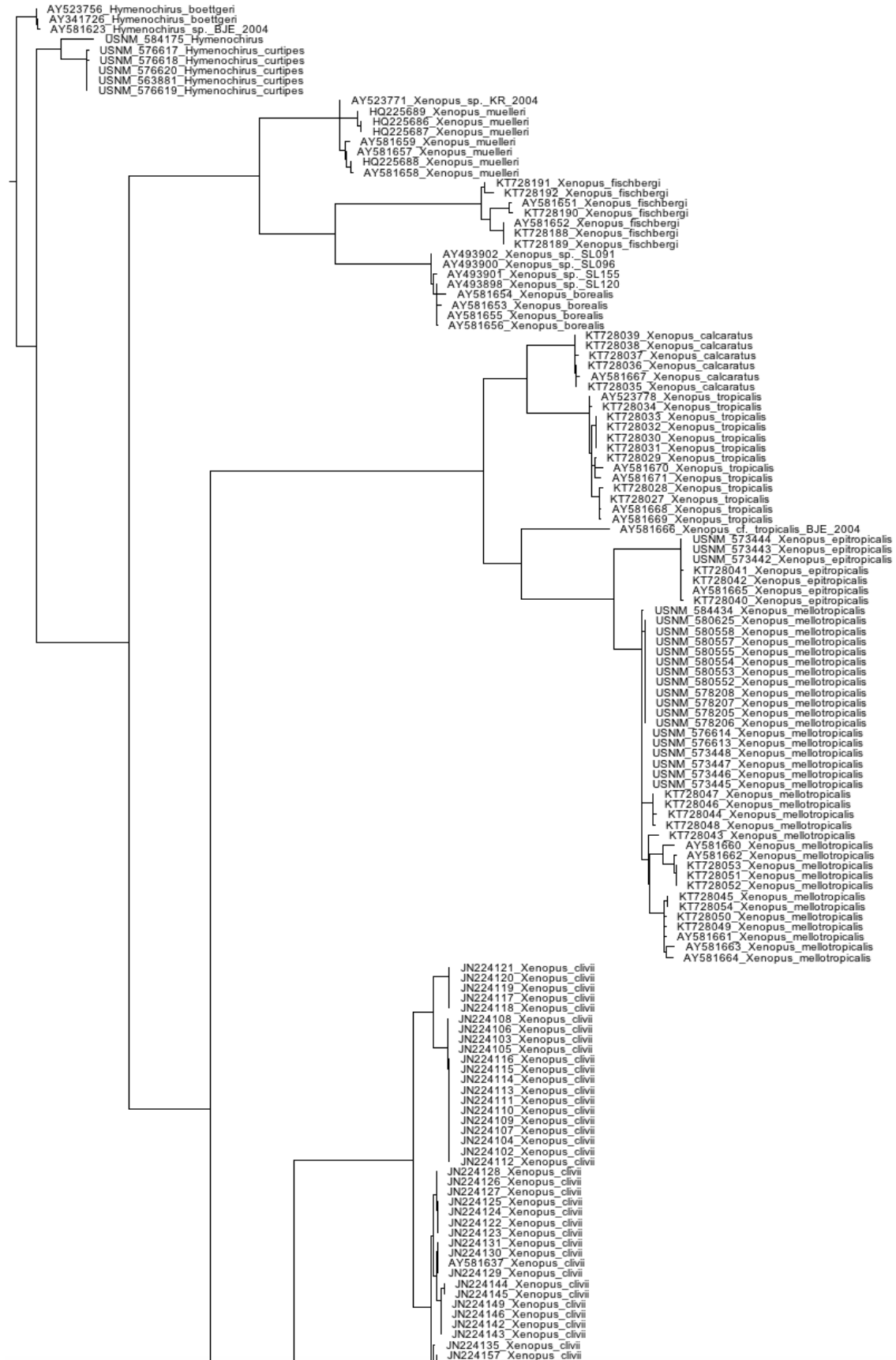

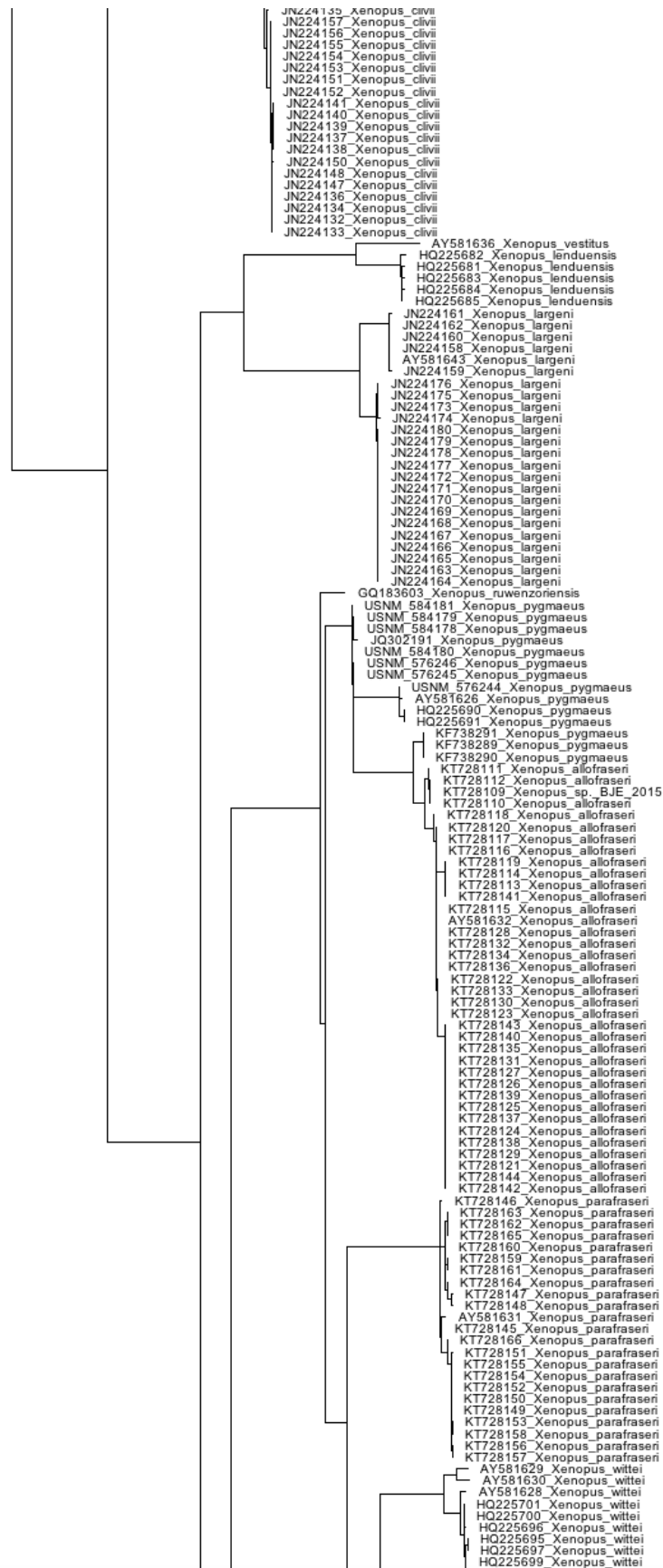

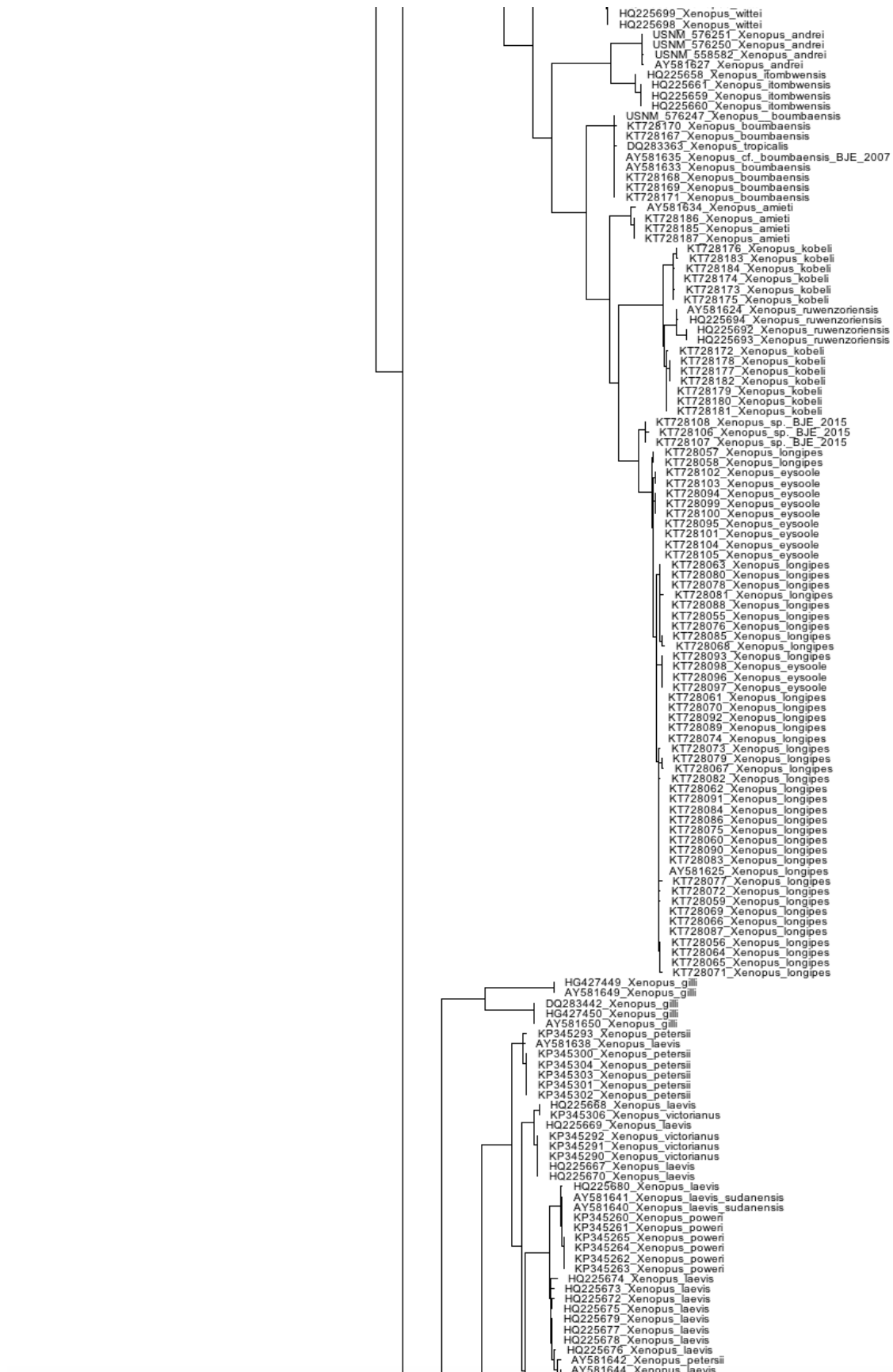

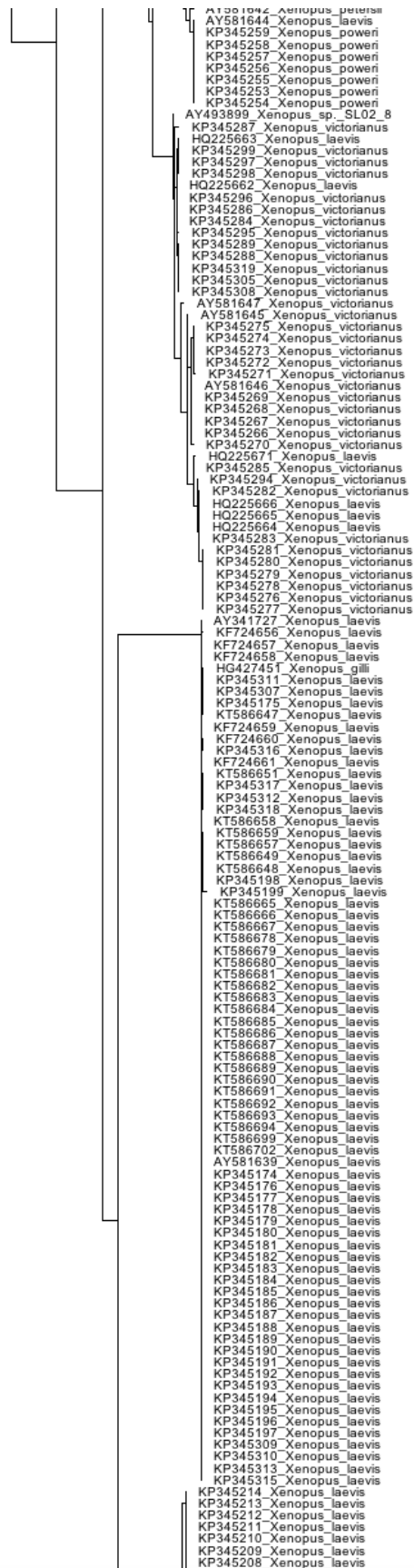

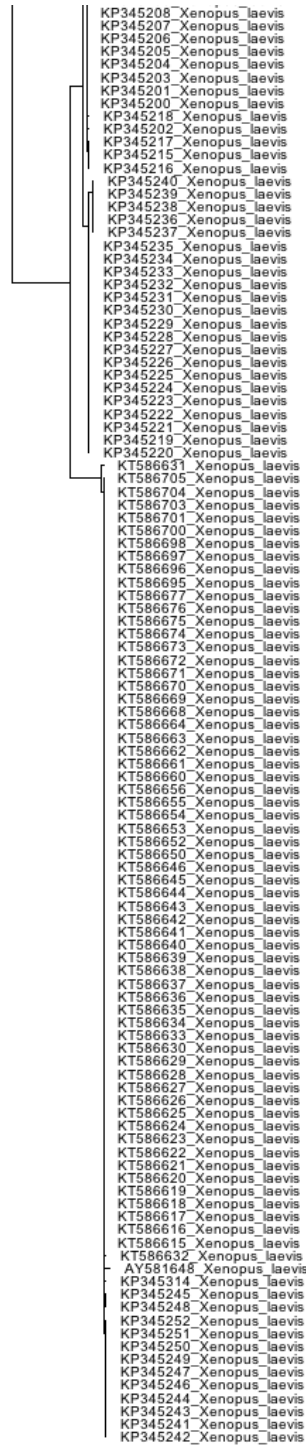

Supplement: S7 Fig — (PDF) [file pone.0187283.s007.pdf]

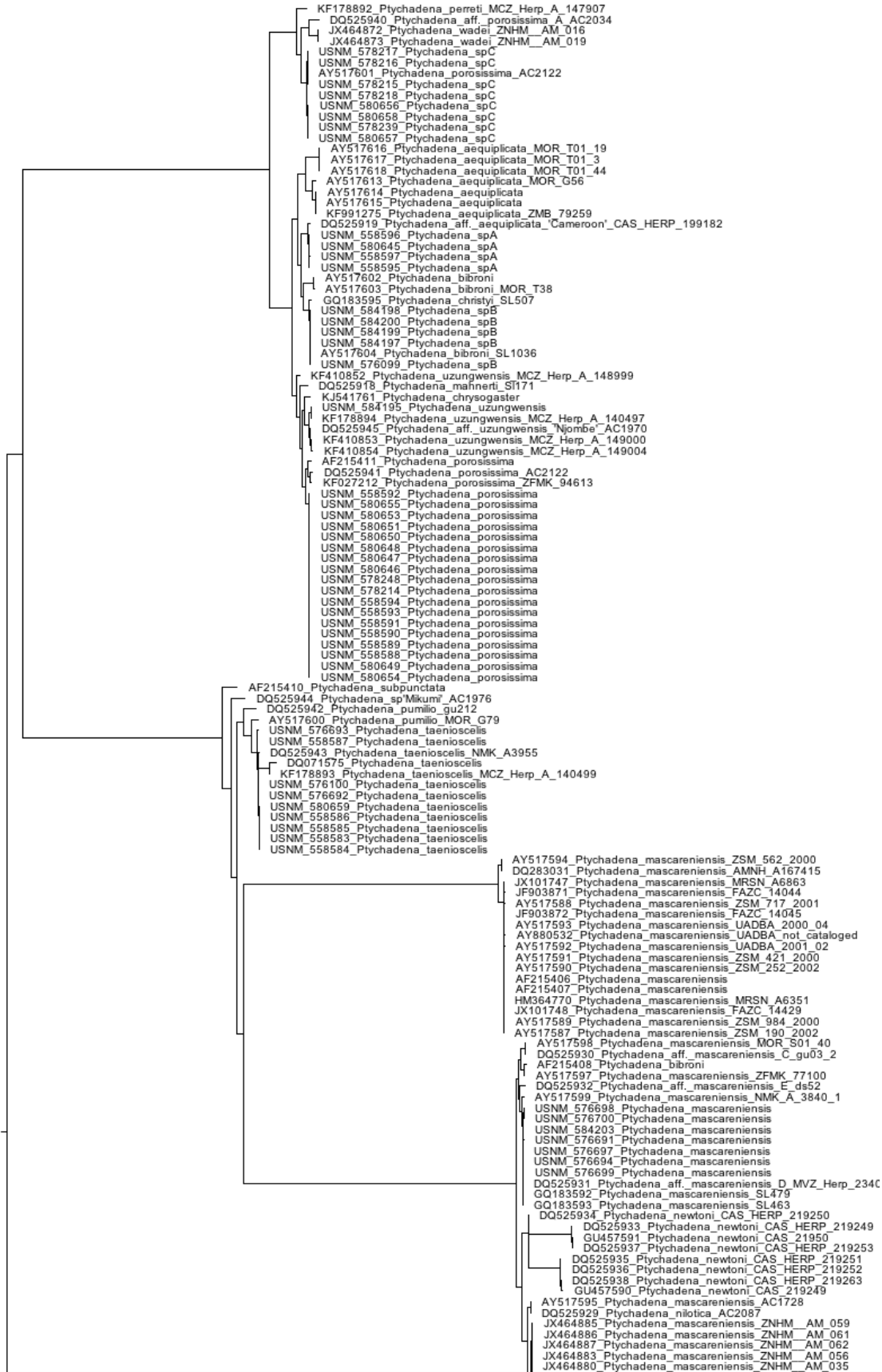

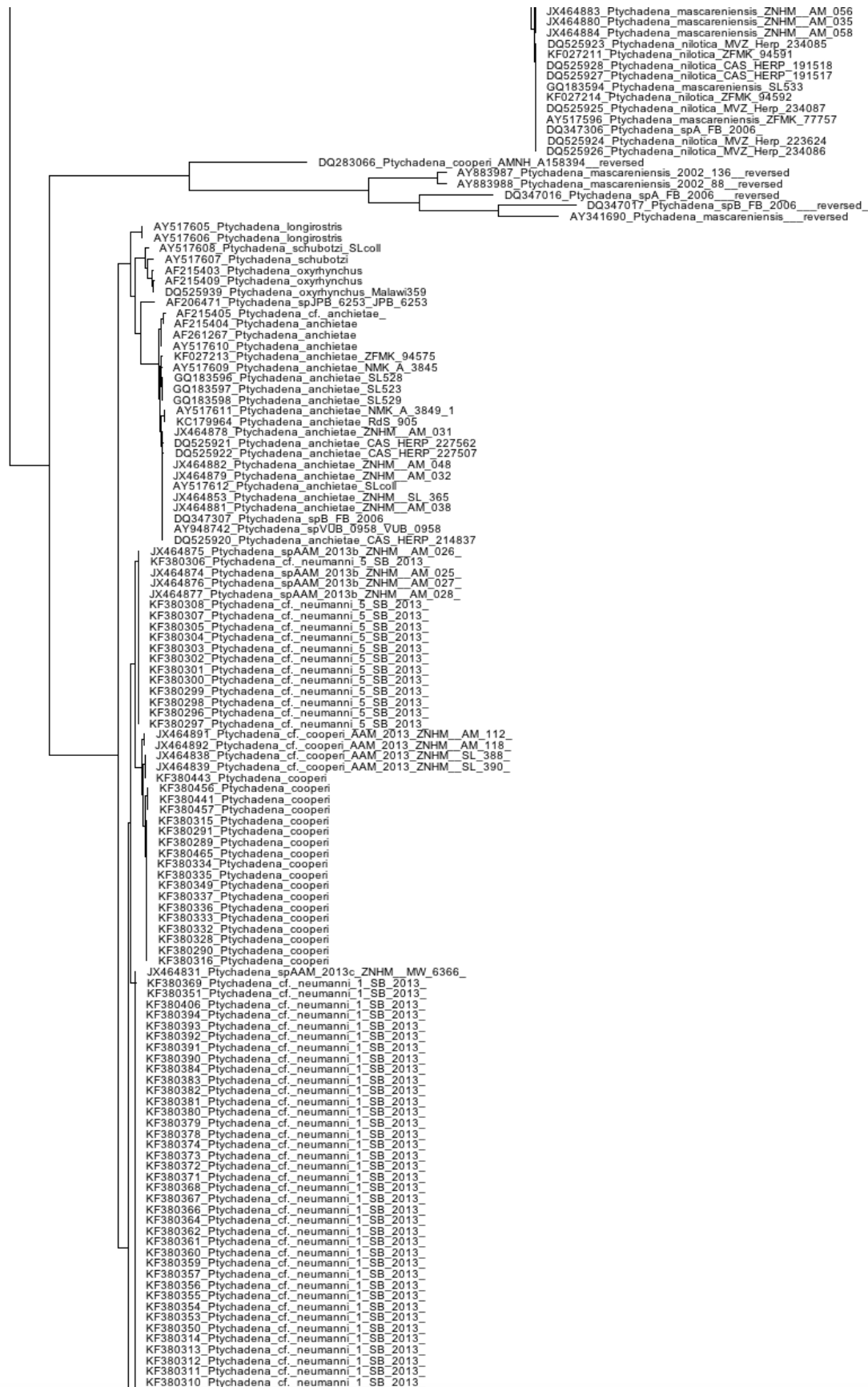

[illegible]

Supplement: S8 Fig — (PDF) [file pone.0187283.s008.pdf]

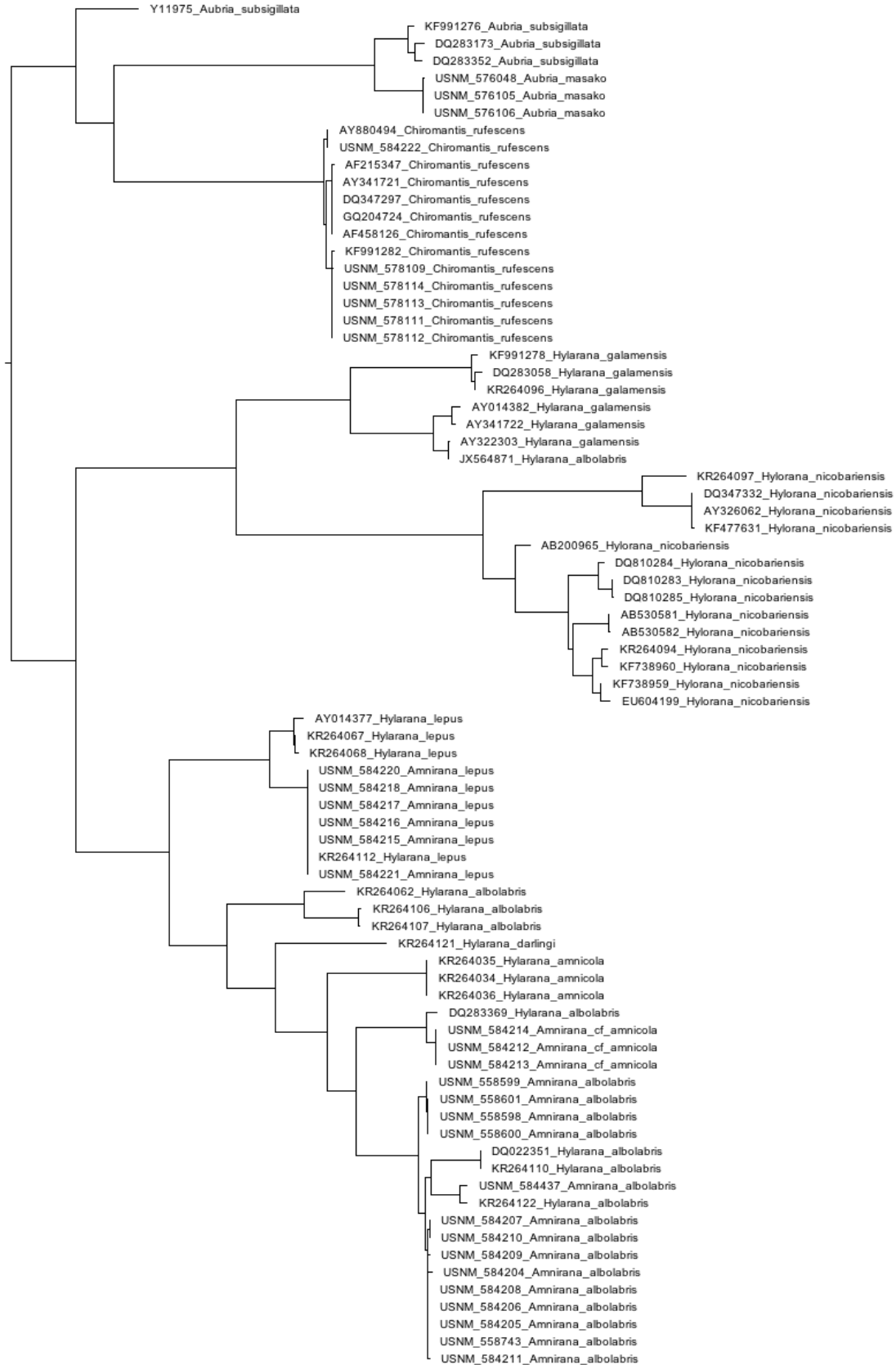

70.0

Supplement: S9 Fig — (PDF) [file pone.0187283.s009.pdf]
